# TPL-2 Regulates Macrophage Lipid Metabolism and M2 Differentiation to Control T_H_2-Mediated Immunopathology

**DOI:** 10.1371/journal.ppat.1005783

**Published:** 2016-08-03

**Authors:** Yashaswini Kannan, Jimena Perez-Lloret, Yanda Li, Lewis J. Entwistle, Hania Khoury, Stamatia Papoutsopoulou, Radma Mahmood, Nuha R. Mansour, Stanley Ching-Cheng Huang, Edward J. Pearce, Luiz Pedro S. de Carvalho, Steven C. Ley, Mark S. Wilson

**Affiliations:** 1 Allergy and Anti-Helminth Immunity Laboratory, The Francis Crick Institute, London, United Kingdom; 2 Mycobacterial Metabolism and Antibiotic Research Laboratory, The Francis Crick Institute, London, United Kingdom; 3 Immune Cell Signaling Laboratory, The Francis Crick Institute, London, United Kingdom; 4 Experimental Histopathology, Mill Hill Laboratory, The Francis Crick Institute, London, United Kingdom; 5 Department of Infection and Immunity, London School of Hygiene and Tropical Medicine, London, United Kingdom; 6 Department of Pathology and Immunology, Washington University School of Medicine, St. Louis, Missouri, United States of America; Uniformed Services University, UNITED STATES

## Abstract

Persistent T_H_2 cytokine responses following chronic helminth infections can often lead to the development of tissue pathology and fibrotic scarring. Despite a good understanding of the cellular mechanisms involved in fibrogenesis, there are very few therapeutic options available, highlighting a significant medical need and gap in our understanding of the molecular mechanisms of T_H_2-mediated immunopathology. In this study, we found that the Map3 kinase, TPL-2 (*Map3k8*; Cot) regulated T_H_2-mediated intestinal, hepatic and pulmonary immunopathology following *Schistosoma mansoni* infection or *S*. *mansoni* egg injection. Elevated inflammation, T_H_2 cell responses and exacerbated fibrosis in *Map3k8* ^–/–^mice was observed in mice with myeloid cell-specific (LysM) deletion of *Map3k8*, but not CD4 cell-specific deletion of *Map3k8*, indicating that TPL-2 regulated myeloid cell function to limit T_H_2-mediated immunopathology. Transcriptional and metabolic assays of *Map3k8* ^–/–^M2 macrophages identified that TPL-2 was required for lipolysis, M2 macrophage activation and the expression of a variety of genes involved in immuno-regulatory and pro-fibrotic pathways. Taken together this study identified that TPL-2 regulated T_H_2-mediated inflammation by supporting lipolysis and M2 macrophage activation, preventing T_H_2 cell expansion and downstream immunopathology and fibrosis.

## Author Summary

Chronic helminth infections can cause significant morbidity and organ damage in their definitive mammalian hosts. Managing this collateral damage can reduce morbidity and preserve vital tissues for normal organ function. One particular consequence of some chronic helminth infections is the deposition of fibrotic scar tissue, following immune responses directed towards helminth material. In this study we tested the role of a particular signalling kinase, TPL-2, and identified that it critically regulated the magnitude of fibrotic scarring following infection. Using several murine models with genetic deletions of TPL-2 in either all cells or specific deletion in subsets of immune cells (*Map3k8* ^–/–^ *Map3k8* ^*fl/fl*^) we identified that expression of TPL-2 in myeloid cells was essential to prevent severe immune-mediated pathology. Using genome-wide analyses and metabolic assays, we discovered that TPL-2 was required for normal lipid metabolism and appropriate activation of myeloid cells / macrophages to limit fibrosis. These results revealed a previously unappreciated role for TPL-2 in preventing severe pathology following infection. Thus, activating this pathway may limit immune mediated pathology following chronic helminth infection. More broadly, this pathway is being targeted to treat inflammatory diseases and cancer [1, 2]. This study would suggest that caution should be taken to prevent untoward co-morbidities and fibrosis-related pathologies in patients when targeting TPL-2.

## Introduction

Immune-mediated pathologies and fibrotic scarring are a major cause of global morbidity and mortality. This is due, in part, to a shortage of available drugs and a lack of novel therapeutic targets to limit fibrogenesis, highlighting a major unmet medical need [3, 4]. Chronic infection resulting in recurring inflammation and wound repair can lead to tissue remodelling, fibrosis and ultimately organ failure. Infection with the parasitic blood fluke, *Schistosoma mansoni*, can cause severe intestinal and hepatic pathologies caused by fibrotic lesions surrounding trapped parasite material. Parasite eggs become lodged within vascularised tissue invoking a distinctive eosinophil and macrophage (MΦ)-rich type-2 immune-mediated granuloma [5]. T_H_2-cell derived IL-4 and IL-13 [6] stimulate IL-4 receptor (IL-4R)-expressing MΦ’s [7, 8] to develop an M2 or alternative activation (AA) state characterised by expression of *Arginase* (*Arg1*), *Resistin-like molecule alpha* (*Retnla*, *Fizz-1*) and *chitinase-like molecules* (*Chi3l3*, *Chi3l4*) [9]. Animal models have indicated that IL-4R-dependent M2-MΦ’s are essential to 1) prevent fatal intestinal damage and sepsis following schistosome infection [7]; 2) orchestrate tissue remodelling and fibrotic responses [10–12] and 3) regulate T_H_2 cell proliferation and activation [13–15]. Despite the clear and well-documented importance of M2-MΦ’s during schistosome infection and the resulting immune-mediated protection, pathology and regulation, the critical regulatory proteins that control M2-MΦ differentiation are poorly understood.

The MAP3 kinase, TPL-2 (also known as COT and encoded by *Map3k8*) is ubiquitously expressed, phosphorylating and activating MEK1/2 following stimulation of Toll-like receptors (TLRs) and the receptors for TNF and IL-1β, leading to the activation of ERK1/2 MAP kinases [16]. TPL-2 is required for T_H_1 and T_H_17-associated inflammation and is essential for the development of autoimmunity and immunity to bacterial and protozoan pathogens [17–20]. In MΦ’s, TPL-2 is required for the synthesis and secretion of a variety of cytokines and chemokine’s following classical activation (CA) with TLR ligands [17, 20–29]. However, it is unclear whether TPL-2 controls M2-MΦ function to regulate chronic T_H_2-associated inflammation and immunopathology.

Two distinct inflammatory pathways contribute to fibrogenic responses; classical, pro-inflammatory type-1/17 and TGF-β-mediated fibrosis [30] and type-2 inflammatory pathways leading to IL-4R-dependent fibrosis [4]. It was recently reported that TPL-2–deficient mice, or inhibition of ERK [31], protected mice from type-1/T_H_17 and TGFβ-mediated pulmonary fibrosis following bleomycin treatment [31] and from hepatic fibrosis following carbon tetrachloride and methionine choline-deficient diet-induced fibrosis [32]. As expected, *Map3k8*–deficient Kupffer cells had reduced TLR-induced IL-1β and pro-fibrotic gene expression, which the authors suggested was responsible for the reduced hepatic fibrosis *in vivo*. However this was not directly tested. Nevertheless, this study raised the possibility that targeting TPL-2 may forestall the progression of hepatic fibrosis. Indeed many small molecule inhibitors have been developed that block TPL-2 signalling in vitro [2], but none have yet made it in to the clinic. However, it is not known whether TPL-2 contributes to chronic type-2 inflammation and IL-4R-mediated fibrosis.

In this study, we used the well-established *Schistosoma mansoni* infection model to test whether TPL-2 regulated chronic type-2 associated inflammation, immunopathology and fibrosis. In contrast to the reduced fibrosis observed in *Map3k8* ^*–/–*^mice following chemical and diet-induced fibrosis [32], *Map3k8* ^*–/–*^mice had significantly increased type-2 immune responses with concomitant elevated inflammation and fibrosis surrounding trapped parasite eggs. Using genome-wide transcriptional analysis and metabolic assays we found that TPL-2 was required for lipid oxidative metabolism and M2-MΦ activation. Specifically, TPL-2 was required for expression of immunoregulatory molecules (*Retnla* and *Arg1*) and regulated pro-fibrotic genes (*Col* genes and *Ctgf*). Consequently, myeloid cell-specific deletion of *Map3k8* resulted in increased type-2 inflammation and significantly increased fibrosis *in vivo*, phenocopying *Map3k8* ^*–/–*^mice. Collectively, our study identifies a novel and previously unappreciated role for TPL-2 as a molecular regulator of lipolysis in M2-MΦ’s, regulating type-2 inflammation, immunopathology and hepatic fibrosis.

## Results

### *Map3k8*-deficient mice develop increased T_H_2-mediated immunopathology and fibrosis following infection with *Schistosoma mansoni*

Following maturation and worm pairing, gravid worms release hundreds of eggs, many of which traverse the wall of the intestine and are released into the environment via the fecal route. However, many eggs do not successfully reach the intestinal lumen but instead become trapped in the intestinal wall or within vascularised organs, particularly the liver. An eosinophil and MΦ-rich fibrotic granuloma forms around trapped eggs causing significant tissue damage, orchestrated by CD4^+^ T_H_2 cells and a highly polarised type-2 immune response [5].

To test whether TPL-2 contributed to *S*. *mansoni*-associated intestinal and hepatic pathology and fibrosis, we infected *Map3k8* ^*–/–*^mice with 50 *S*. *mansoni* cercariae.

**Fig 1:**
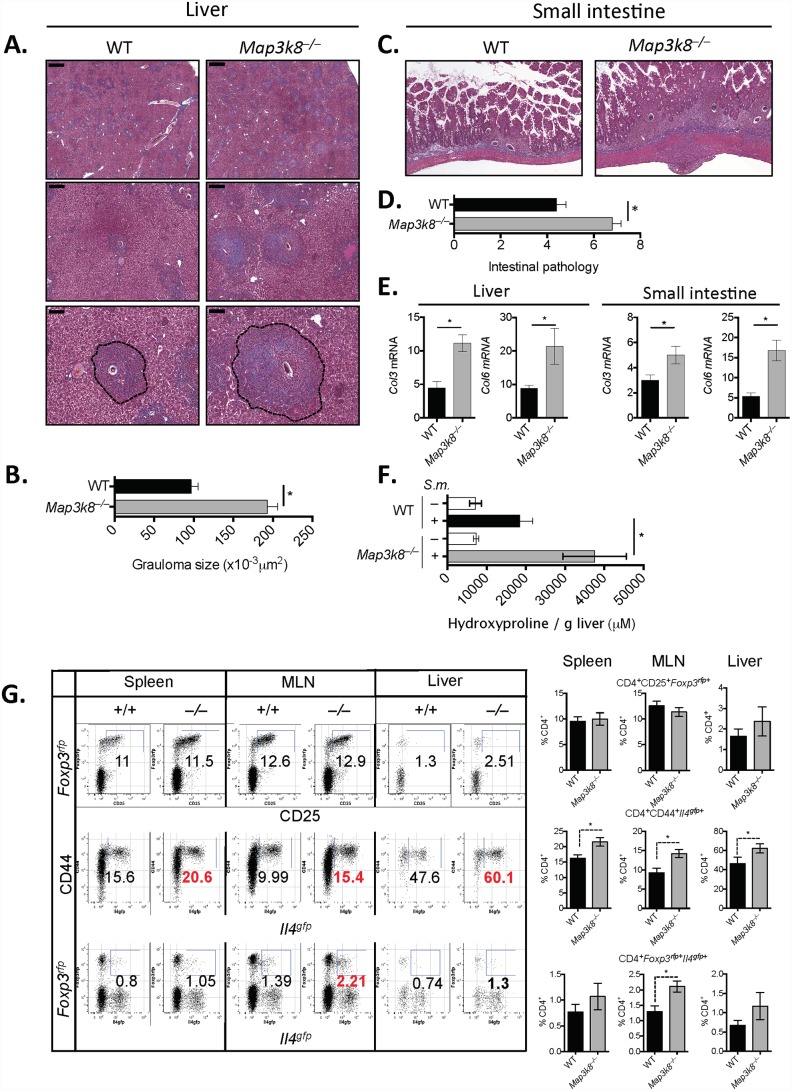
*Map3k8* ^–/–^mice develop increased hepatic and intestinal inflammation and fibrosis following *S*. *mansoni* infection. WT and *Map3k8* ^*–/–*^mice were infected percutenously with 50 *S*. *mansoni* cercariae and analysed at 8 weeks post-infection. A & C) Perfused tissue was fixed and embedded in paraffin before sectioning and staining with Masson’s trichrome. B) Granuloma size was determined from 10–20 individual granulomas per sample measured using Image J. Scale bars are 1000μm (top), 200μm (middle) and 100μm (bottom). D) Intestinal pathology score, as described in methods. E) Expression of *Col3* and *Col6* was determined from RNA extracted from liver or small intestinal tissue. Data is expressed relative to HPRT. F) Hydroxyproline was quantified in liver tissue from naïve and infected animals. G) Frequency of T_REG_ (CD4^+^CD25^+^ *Foxp3* ^*RFP*+^) and T_H_2 (CD4^+^CD44^+^ *Il4* ^*GFP*+^) cells in the spleen, mesenteric lymph nodes (MLN) and liver were determined by FACS. All experiments are representative of 2–3 independent experiments with 5–10 mice/genotype. * p< 0.05 as assessed by two-tailed Mann-Whitney test.

Histological analysis indicated that *S*. *mansoni-*infected *Map3k8* ^*–/–*^mice had more fibrosis in the liver with larger hepatic granulomas (Fig 1A and 1B), despite a similar egg burden (S1 Fig) and serum LPS level as *S*. *mansoni-*infected WT mice (S1 Fig). Similarly, intestinal inflammation was also significantly increased in *Map3k8* ^*–/–*^mice (Fig 1C and 1D). Consistent with the increased collagen staining observed in *Map3k8* ^*–/–*^mice, collagen-synthesising genes, *Col3* and *Col6*, were both significantly elevated in the liver and small intestine of *Map3k8* ^*–/–*^mice, compared to WT controls (Fig 1E), with significantly more hydroxyproline in the liver of *Map3k8* ^*–/–*^mice (Fig 1F). *Map3k8* ^*–/–*^mice had elevated expression of *Il13* in the liver, but not *Il1b*, *Tgfb*, *Il17a*, *Ifng*, *Tnfa* or *Il6* (S1 Fig), suggesting that IL-13-driven fibrosis was exacerbated in *Map3k8* ^*–/–*^mice [33] rather the development of other inflammatory mechanisms of fibrosis [30].

CD4^+^ T_H_2 cell-derived IL-4 and IL-13 are essential for granuloma formation [6], mobilising and activating a suite of innate immune cells, including MΦ’s and eosinophils, and promoting local collagen deposition. T_H_2 cell-mediated inflammatory responses are controlled by Foxp3^+^ regulatory T (T_REG_) cells [34], which restrain T_H_2 cell expansion. It was previously suggested that T cell intrinsic TPL-2 regulates T_H_2 [35] and Foxp3^+^ T_REG_ cell differentiation [36]. However, these conclusions were based on *in vitro* experiments and were not tested *in vivo*. To determine whether *Map3k8* ^–/–^mice had dysregulated T_H_2 and Foxp3^+^ T_REG_ responses following *S*. *mansoni* infection, we crossed *Map3k8* ^*–/–*^mice with *Il4* ^*gfp*^ and *Foxp3* ^*rfp*^ reporter mice, generating dual-reporter *Map3k8* ^*–/–*^mice (*Map3k8* ^*–/–*^ *Foxp3* ^*rfp*^ *Il4* ^*gfp*^). These reporter mice allowed us to accurately and simultaneously monitor T_H_2 (*Il4* ^*gfp*+^) and Foxp3^+^ T_REG_ (*Foxp3* ^*rfp*+^) cells in *Map3k8* ^*–/–*^mice without the requirement for re-stimulation or intra-nuclear staining. *Map3k8*-deficiency did not alter CD4^+^CD25^+^ *Foxp3* ^*rfp*+^ T_REG_ cell frequencies in the spleen, mesenteric lymph node (MLN) or in the local liver tissue, indicating that TPL-2 was not required for T_REG_ cell development or recruitment following *S*. *mansoni* infection (Fig 1G, top row). However, CD4^+^CD44^+^ *Il4* ^*gfp+*^ T_H_2 cells in both lymphoid tissues and the liver were significantly increased in *Map3k8* ^*–/–*^mice compared to WT mice (Fig 1G, middle row). *Map3k8*-deficiency also increased the frequency of *Il4* ^*gfp+*^ *Foxp3* ^*rfp+*^ cells in the MLN.

Pharmacological inhibition of MEK1/2, a downstream target of TPL-2, protected mice from bleomycin induced fibrosis [31]. We have previously reported that bleomycin-induced fibrosis is mediated by a pro-inflammatory type-1/type-17 and TGFβ driven response, distinct from type-2 mediated pulmonary fibrosis[30]. It therefore remained unclear whether TPL-2 contributed to type-2 driven pulmonary fibrosis. To test this we treated mice intravenously with *S*. *mansoni* eggs to invoke type-2 inflammation in the lungs leading to the development of pulmonary fibrosis, as previously described [30]. Similar to responses in the liver, *Map3k8* ^–/–^mice had increased collagen staining in the lung and increased hydroxyproline levels, compared to WT mice given *S*. *mansoni* eggs (S2 Fig). In the lung tissue and local draining thoracic lymph nodes (TLN), *Map3k8* ^–/–^mice had increased Th2 cell frequency (S2 Fig) promoting increased Il13, Col6 and Mmp12 expression in the lung (S2 Fig). Collectively, these data indicate that TPL-2 is an important negative regulator of type-2 inflammation, immunopathology and fibrosis following *S*. *mansoni* infection or *S*. *mansoni* egg induced pulmonary fibrosis *in vivo*.

### T cell-intrinsic TPL-2 does not regulate T_H_2-mediated immunopathology following *S*. *mansoni* infection

**Fig 2:**
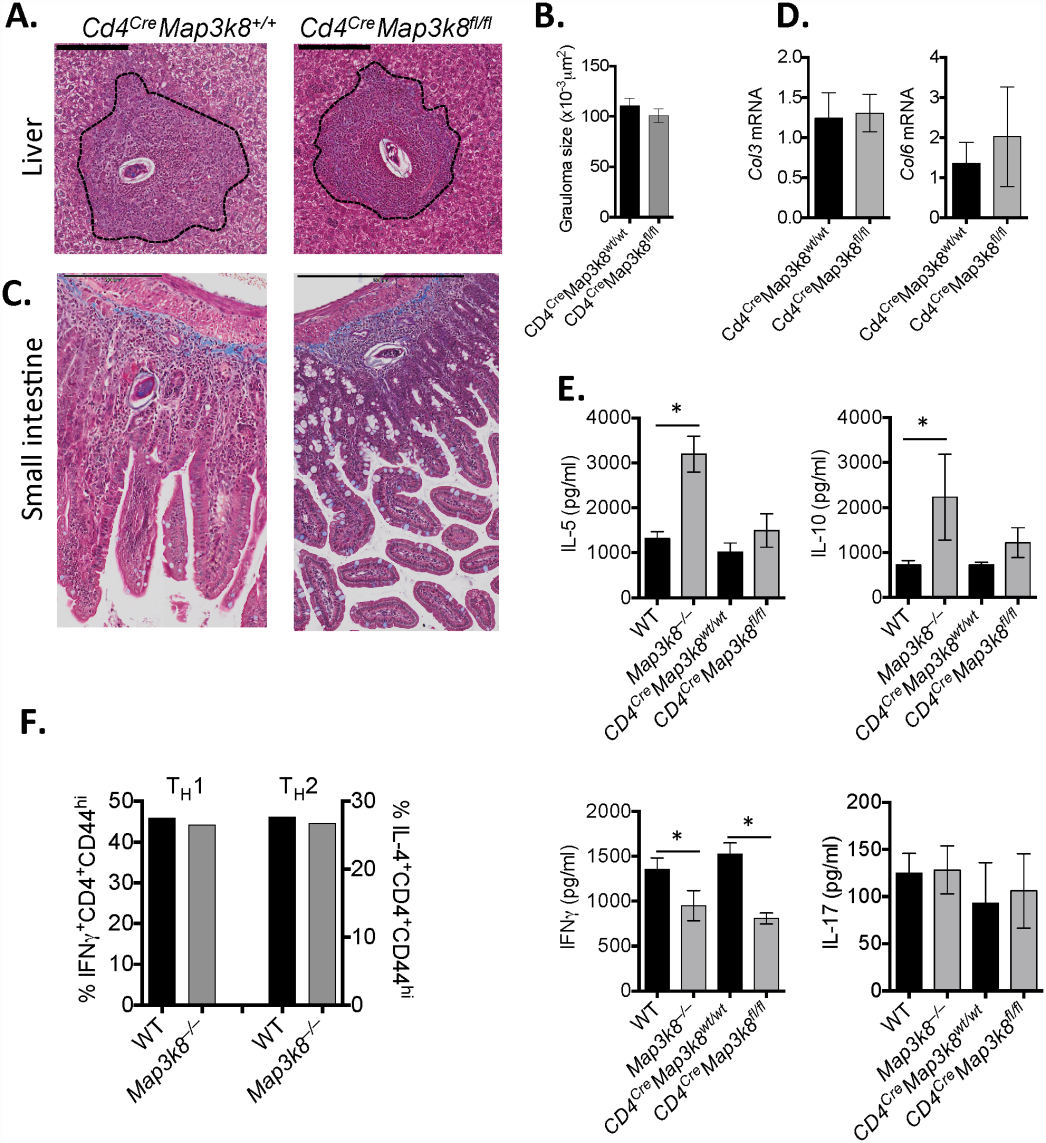
T cell-intrinsic *Map3k8* does not contribute to exacerbated inflammation and pathology following *S*. *mansoni* infection. *Cd4* ^*Cre*^ *Map3k8* ^*+/+*^ and *Cd4* ^*Cre*^ *Map3k8* ^*fl/fl*^ mice were infected percutenously with 50 *S*. *mansoni* cercariae and analysed at 8 weeks post-infection. A–C) Perfused tissue was fixed and embedded in paraffin before sectioning and staining with Masson’s trichrome. B) Granuloma size was determined from 10–20 individual granulomas per sample measured using Image J. D) Expression of *Col3* and *Col6* was determined from RNA extracted from liver or small intestinal tissue. Data is expressed relative to HPRT and shown as a fold-change relative to uninfected mice. E) Mesenteric lymph node cells were re-stimulated with anti-CD3 for 3 days. Cytokines were measured in supernatants, by ELISA. F) Naive T cells (CD4^+^CD44^−^CD25^−^CD62L^+^) were FACS purified from WT and *Map3k8* ^*–/–*^mice and cultured under T_H_1 and T_H_2 conditions. Frequencies of CD44^+^IFNγ^+^ and CD44^+^IL-4^+^ cells were determined by intracellular FACS analysis on day 7. All experiments are representative of 2–3 independent experiments with 5–10 mice/genotype. * p< 0.05 as assessed by two-tailed Mann-Whitney test.

It has previously been reported that T cell-intrinsic TPL-2 regulates T_H_2 cell differentiation *in vitro* and acute type-2 inflammation in the airways [35], however it has remained unclear whether T cell-intrinsic TPL-2 regulates T_H_2 cell differentiation and function *in viv*o. To formally test whether T cell-intrinsic TPL-2 contributed to the enhanced inflammation and fibrosis observed in *Map3k8* ^–/–^mice (Fig 1) we restricted *Map3k8* deficiency to T cells using *Cd4* ^*Cre*^ *Map3k8* ^*fl/fl*^ mice. Deletion of *Map3k8* in T cells (*Cd4* ^*Cre*^ *Map3k8* ^*fl/fl*^) had no impact on granuloma development in the liver (Fig 2A and 2B) or small intestine (Fig 2C) following *S*. *mansoni* infection. Similarly, fibrosis (Fig 2A and 2C) and expression of collagen synthesising genes, *Col3* and *Col6*, were not affected following the deletion of *Map3k8* in CD4^+^ cells (Fig 2D). IL-5 and IL-10 production was significantly increased in re-stimulated MLN cells from *Map3k8* ^–/–^mice, compared to WT cells; however production of these cytokines was not affected when *Map3k8* was deleted in T cells only (Fig 2E). IL-17 production was low and unchanged between all groups, however IFNγ secretion from lymph node cells was reduced in *Map3k8* ^–/–^mice and *Cd4* ^*Cre*^ *Map3k8* ^*fl/fl*^ mice, in line with a previous report [18]. To further test whether T cell intrinsic TPL-2 was required for T_H_2 cell differentiation, we isolated naïve T cells (TCRβ^+^CD4^+^CD44^_^) from WT and *Map3k8* ^–/–^mice and polarised them under T_H_1 or T_H_2 conditions *in vitro*. Similar frequencies of IFNγ^+^ or IL-4^+^ cells were observed between WT and *Map3k8* ^–/–^T cells, respectively (Fig 2F), suggesting that T cell-intrinsic TPL-2 does not contribute to T_H_1 or T_H_2 differentiation *in vitro*. Taken together, these data indicate that T cell-intrinsic TPL-2 is required for optimal IFNγ secretion *in vivo*, but does not contribute to T_H_2 cell differentiation *in vitro* or *in vivo*, and that T cell-intrinsic TPL-2 does not contribute to T_H_2 cell-mediated immunopathology following *S*. *mansoni* infection.

### Myeloid cell-intrinsic *Map3k8* critically regulates T_H_2-mediated immunopathology

**Fig 3:**
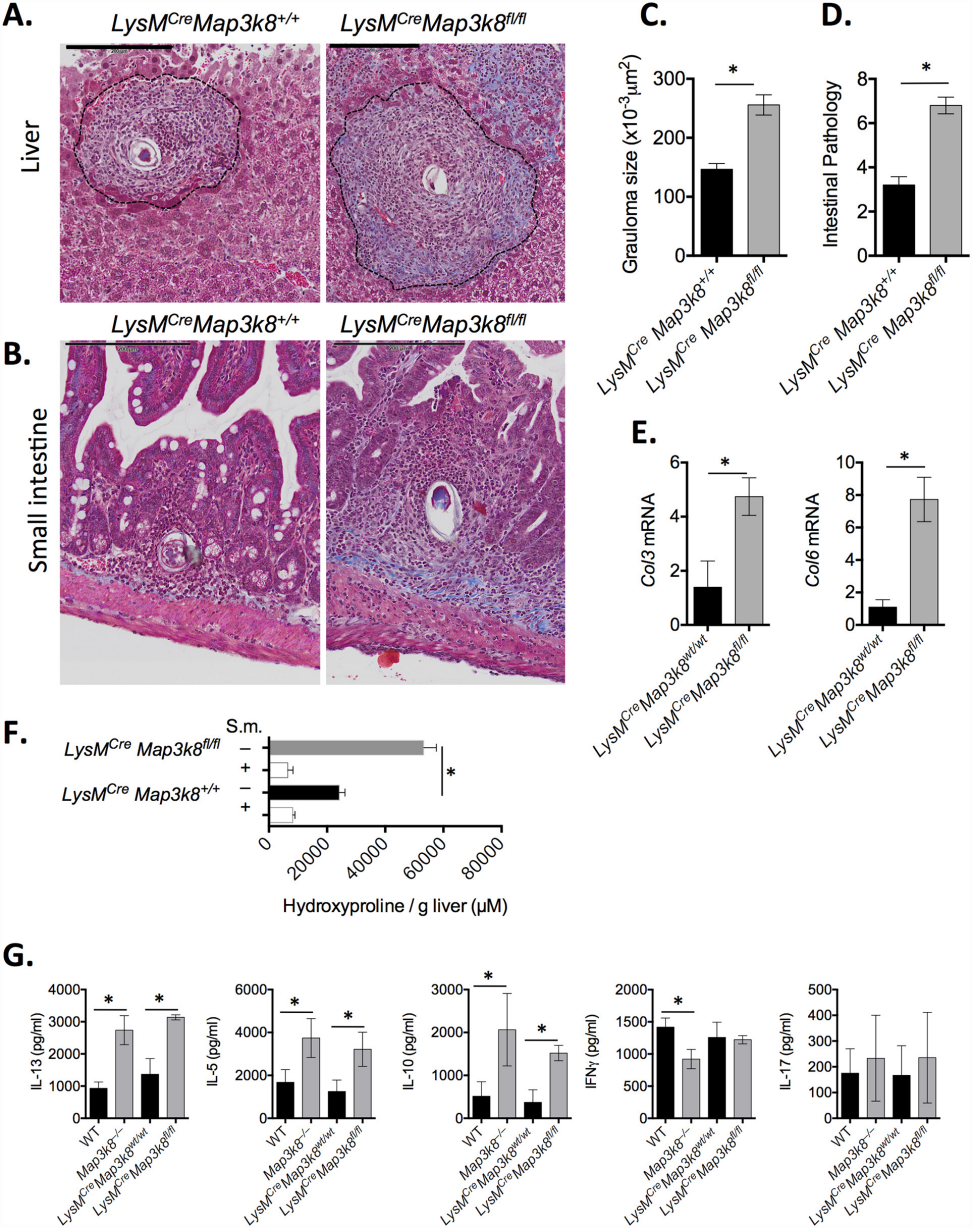
Myeloid cell (*LysM* ^+^) expression of Map3k8 regulates T_H_2-mediated immunopathology and fibrosis following *S*. *mansoni* infection. *LysM* ^*Cre*^ *Map3k8* ^*+/+*^ and *LysM* ^*Cre*^ *Map3k8* ^*fl/fl*^ mice were infected percutenously with 50 *S*. *mansoni* cercariae and analysed at 8 weeks post-infection. A–B) Perfused tissue was fixed and embedded in paraffin before sectioning and staining with Masson’s trichrome. Scale bar is 200μm. C) Granuloma size was determined from 10–20 individual granulomas per sample measured using Image J. D) Intestinal pathology score, as described in methods. E) Expression of *Col3* and *Col6* was determined from RNA extracted from liver. Data is expressed relative to HPRT and presented as a fold-change relative in infected WT mice. F) Hydroxyproline was quantified in liver tissue from naïve and infected animals. G) Mesenteric lymph node cells were re-stimulated with anti-CD3 for 3 days. Cytokines were measured in supernatants, by ELISA. All experiments are representative of 2–3 independent experiments with 5–10 mice/genotype. * p< 0.05 as assessed by two-tailed Mann-Whitney test.

Alternatively activated macrophages (AA or M2-MΦ) contribute significantly to inflammation, immunopathology and fibrosis following *S*. *mansoni* infection [12]. TPL-2 has a well-defined role in classically activated MΦ’s (M1 or CA-MΦ) [17, 20–29], however it is unclear whether TPL-2 contributes to M2-MΦ following *S*. *mansoni* infection. Firstly, to test whether myeloid cell-intrinsic TPL-2 contributed to the exacerbated immunopathology observed in *Map3k8* ^–/–^mice, we restricted *Map3k8* deletion to Lysozyme M-expressing cells using *LysM* ^*Cre*^ *Map3k8* ^*fl/fl*^ mice (S3 Fig). Mice with myeloid cell-specific deletion of *Map3k8* had significantly more inflammation with larger hepatic (Fig 3A and 3C) and intestinal (Fig 3B) granulomas and more severe intestinal pathology (Fig 3D), without any appreciable change in serum LPS (S3 Fig). Of note, a distinct collagen-rich fibrotic ring surrounded hepatic granulomas in *LysM* ^*Cre*^ *Map3k8* ^*fl/fl*^ mice, which was absent in mice with WT myeloid cells. Increased collagen staining in the liver was supported by increased expression of collagen-synthesising genes, *Col3* and *Col6* (Fig 3E) and increased hydroxyproline (Fig 3F). Similar to *Map3k8* ^*–/–*^mice, mice with myeloid cell-specific deletion of *Map3k8* had elevated type-2 cytokine secretions (IL-13, IL-5 and IL-10) following lymph node re-stimulation without any appreciable change in IFNγ or IL-17A secretion (Fig 3E). Similarly, elevated expression of *Il13* but not *Il1b*, *Tgfb*, *Il17a*, *Ifng*, *Tnfa* or *Il6* was observed in *LysM* ^*Cre*^ *Map3k8* ^*fl/fl*^ mice, compared to control mice (S3 Fig). These data clearly indicated that macrophage/myeloid cell intrinsic-TPL-2 contributed significantly to the regulation of T_H_2-mediated inflammation and fibrosis following *S*. *mansoni* infection.

### *Map3k8* regulates M2 macrophage activation

**Fig 4:**
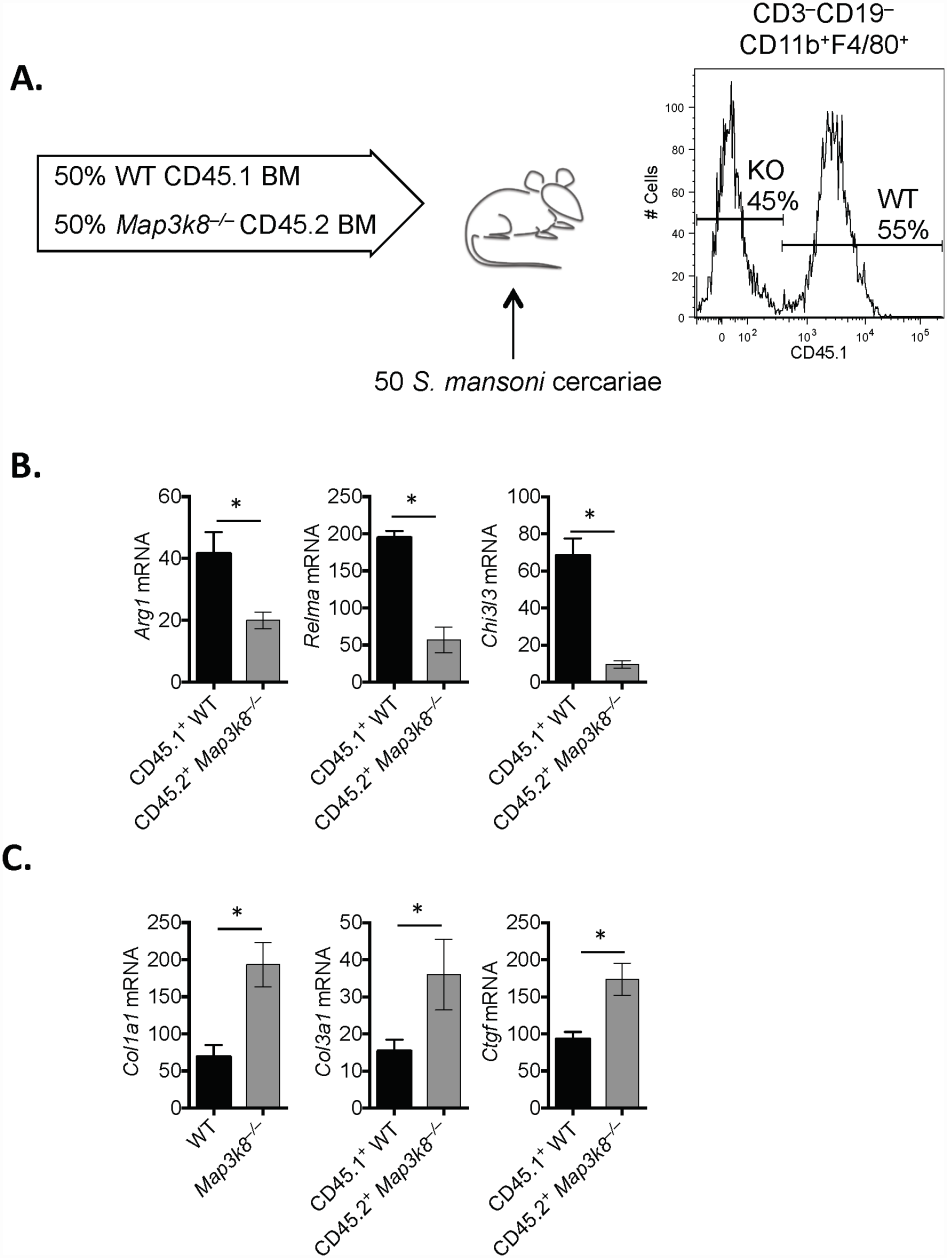
TPL-2 is required for M2 activation of Macrophages, *in vivo*. WT C57BL/6 mice were lethally irradiated (900rad) and reconstituted with 50% CD45.1+ WT bone marrow and 50% CD45.2+ *Map3k8* ^–/–^bone marrow and left for 6–8 weeks, prior to infection with 50 *S*. *mansoni* cercariae. A) After 8 weeks of infection, mice were sacrificed and CD3^−^CD19^−^CD11b^+^F4/80^+^ Macrophages were FACS-sorted. B-C) Expression of *Arg1*, *Relma*, *Chi3l3*, *Col1a1*, *Col3a1* and *Ctgf* was determined from RNA extracted from purified macrophages. Data is expressed relative to HPRT and presented as a fold-change relative in genotype-controlled naïve bone marrow derived macrophages. Experiments are representative of 2 independent experiments with 5 mice/genotype. * p< 0.05 as assessed by two-tailed Mann-Whitney test.

T_H_2-cell derived IL-4 and IL-13 [6] activates IL-4 receptor (IL-4R)-expressing MΦ’s [7, 8] to prevent lethal pathology following *S*. *mansoni* infection. To determine whether myeloid cell-intrinsic TPL2 contributed to M2-MΦ’s activation *in vivo*, we isolated MΦ’s ex vivo from infected mice. Chimeric mice were generated following the observation that *Map3k8* ^–/–^mice and *LysM* ^*Cre*^ *Map3k8* ^*fl/fl*^ mice had significantly elevated type-2 inflammation and fibrosis, compared to WT controls (Figs 1 and 3). Generating 50:50 chimeric mice by reconstituting lethally irradiated WT mice with 50% bone marrow from CD45.1^+^WT mice and 50% bone marrow from CD45.2^+^ *Map3k8* ^–/–^mice (Fig 4A), normalised and controlled for these environmental differences allowing us to more accurately compare WT and *Map3k8* ^–/–^MΦ’s *ex vivo*. Following 8-weeks of *S*. *mansoni* infection, CD45.1^+^WT or CD45.2^+^ *Map3k8* ^–/–^CD3^−^CD11b^+^F4/80^+^CD11b^+^ MΦ’s were FACS sorted for analysis (Fig 4A). *Map3k8* ^–/–^MΦ’s had significantly lower expression of *Arg1*, *Relma* and *Chi3l3*, compared to WT MΦ’s isolated form the same tissue (Fig 4B). In addition, *Map3k8* ^–/–^MΦ’s had elevated expression of collagen synthesising genes (*Col1*, *Col3*) and connective tissue growth factor (Ctgf). These data indicate that macrophage cell-intrinsic TPL2 was required for M2-MΦ activation and regulated expression of pro-fibrotic genes.

**Fig 5:**
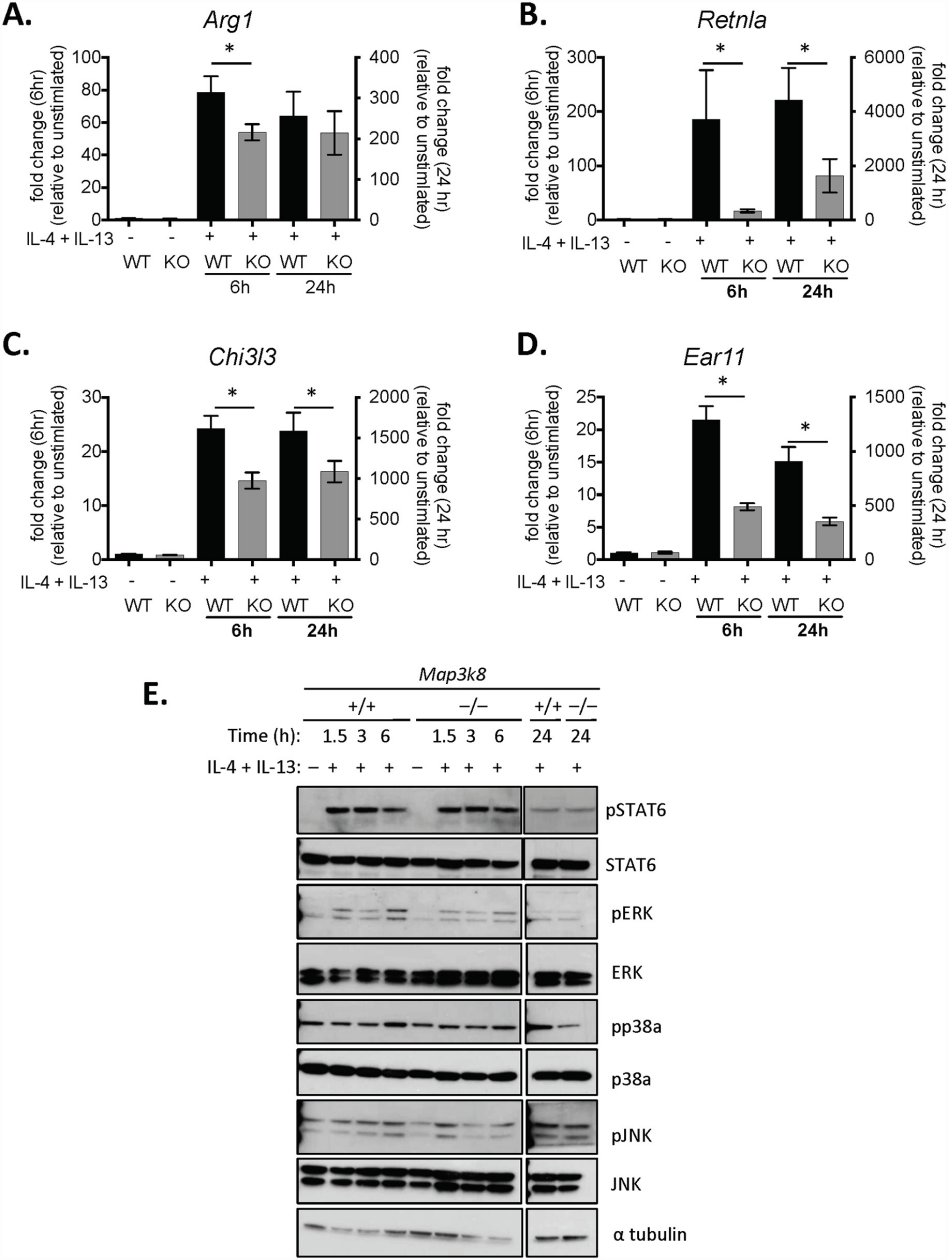
TPL-2 is required for M2 activation of Macrophages, *in vitro*. A-D) Bone marrow-derived macrophages (BMDM) were stimulated with IL-4 and IL-13 for 6 or 24 hours, as indicated. Cells were harvested, RNA extracted and gene expression was determined by qRT—PCR and expressed relative to un-stimulated genotype control cells. E) BMDM’s were stimulated for 1.5, 3 and 6 hours, as indicated. Total Protein was extracted with phosphorylated and total protein levels of STAT6, ERK, p38a, JNK and α-tubulin determined by western blot. All experiments are representative of 2–3 independent experiments with 3–5 biological replicates and 3 technical replicates in each experiment. * p< 0.05 as assessed by two-tailed Mann-Whitney test.

To determine how TPL-2 was regulating M2-MΦ activation we used the well-described *in vitro* macrophage activation assay, activating bone marrow-derived MΦ’s (BMDM) with IL-4 and IL-13. Following 6hrs of exposure to IL-4 and IL-13, *Arg1*, *Retnla*, *Chi3l3* and *Ear11* were all significantly reduced in *Map3k8* ^–/–^ M2-MΦ’s, compared to WT M2-MΦ’s (Fig 5A–5D), similar to that observed in ex vivo MΦ’s. At 24hrs, *Retnla*, *Chi3l3* and *Ear11* were still significantly reduced in *Map3k8* ^–/–^ M2-MΦ’s, demonstrating a non-redundant role for TPL-2 in M2-MΦ activation. The early reduction of *Arg1* expression in *Map3k8* ^–/–^ M2-MΦ’s led to a reduction in arginase activity with reduced ornithine production, as determined by LCMS (S4 Fig). Inhibition of the kinase activity of TPL-2, using the pharmacological inhibitor, C34 [37], phenocopied *Map3k8* ^–/–^MΦ’s indicating that the kinase activity of TPL-2 was responsible for the reduced *Retnla* expression in M2-MΦ (S4 Fig). Phosphorylated (p)STAT6, pERK, pp38α and pJNK were similar in *Map3k8* ^–/–^and WT MΦ’s (Fig 5E), suggesting that TPL-2 did not regulate responsiveness of MΦ’s to IL-4 and/or IL-13 and was not required for activation of these downstream transcription factors or kinases.

**Fig 6:**
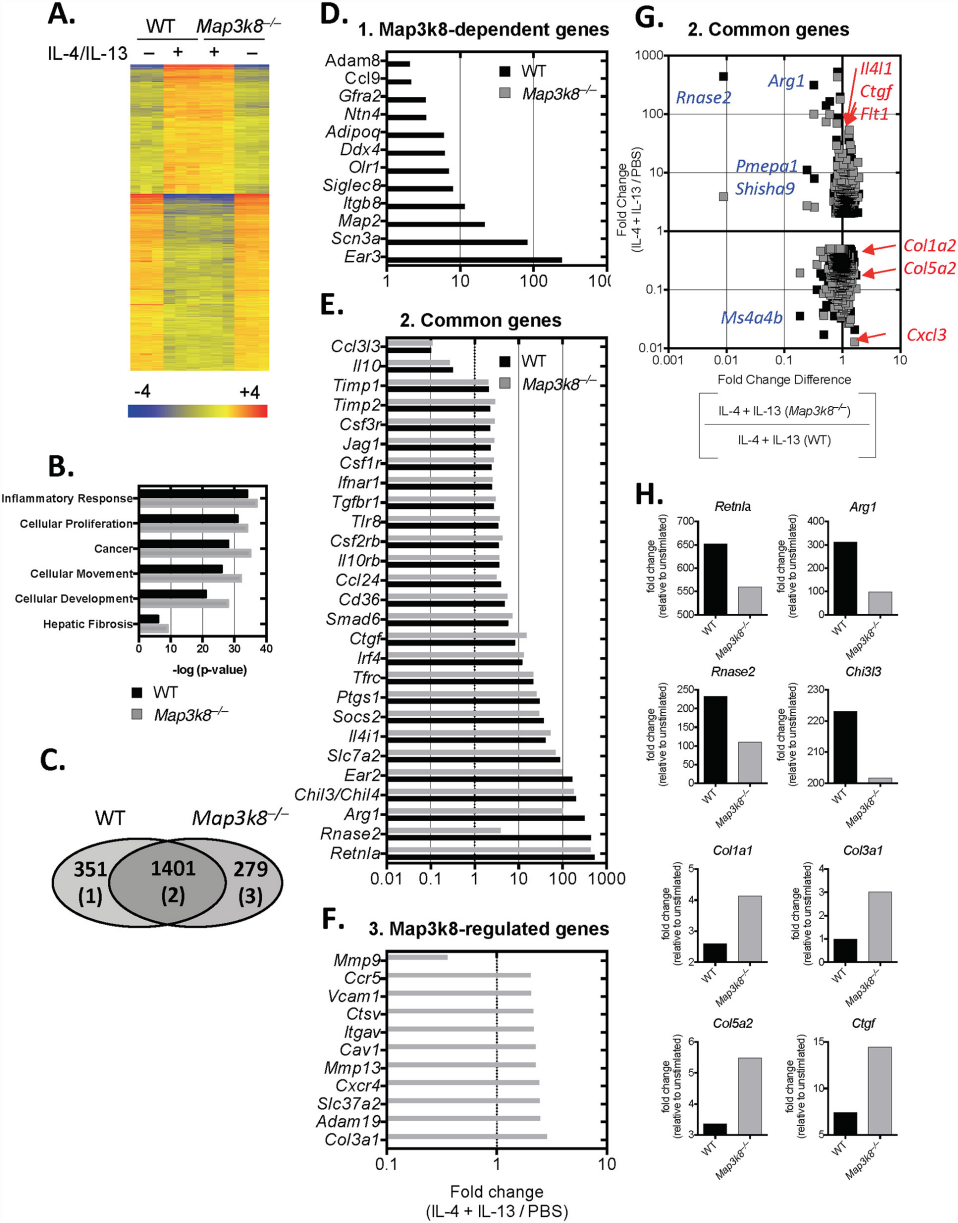
TPL-2 regulates pro-fibrotic and immuno-regulatory pathways in M2 macrophages. Bone marrow-derived macrophages (BMDM) were stimulated with IL-4 and IL-13 for 24 hours. Cells were harvested, RNA extracted and genome-wide transcriptional expression was determined by microarray analysis using 3 biological replicates. A) Heat map of differentially regulated genes in un-stimulated and IL-4+IL-13 stimulated cells. B) Ingenuity pathways analysis of transcriptional profiles of differentially regulated genes. C-F) Venn diagram and bar graphs of TPL-2 dependent (1), common (2) and TPL-2-regulated genes (3). G and H) Ratio of Ratios graph (top) and bar graphs (H) showing increased pro-fibrotic (red) and decreased Immunoregulatory (blue) genes in *Map3k8* ^*–/–*^macrophages, relative to WT macrophages (x-axis) and un-stimulated macrophages (y-axis).

To determine whether TPL-2 regulated the expression of other genes, beyond the characteristic M2-MΦ-associated genes, we profiled the transcriptional landscape of WT and *Map3k8* ^*–/–*^MΦ’s following 24hrs of IL-4 and IL-13 stimulation (Fig 6A). Pathway analysis identified increased inflammatory pathways in *Map3k8* ^*–/–*^ M2-MΦ’s, including proliferation, migration and fibrogenesis (Fig 6B). Of the significantly differentially regulated genes (P<0.05, > 2-fold, relative to un-stimulated) (Fig 6C), we identified 351 *Map3k8*-dependent genes (i.e. genes differentially regulated in WT only, Fig 6C and 6D) and 279 *Map3k8*-regulated genes (i.e. genes differentially regulated in *Map3k8* ^*–/–*^MΦ’s only, Fig 6C and 6F). Of note, several of these elevated genes in *Map3k8* ^*–/–*^ M2-MΦ’s contribute to fibrogenesis, including *Adam19* [38], *Cxcr4* [39], *Mmp13* [40], *Cav1* [41], *Itgav* [42] and *Vcam1* [43] (Fig 6F). Of the commonly regulated genes in both WT and *Map3k8* ^*–/–*^ M2-MΦ’s (Fig 6E and 6G) *Map3k8* ^*–/–*^ M2-MΦ’s had elevated expression of collagen-synthesising genes (*Col1a1*, *Col3a1*, *Col5a2*) and the connective tissue growth factor, *Ctgf* (Fig 6H) compared to WT MΦ’s (Fig 6G). Concurrent with increased expression of pro-fibrotic genes, characteristic M2-MΦ genes (*Retnla*, *Arg1*, *Ear11* (*Rnase2*) and *Chi3l3*) were reduced, compared to WT M2-MΦ’s (Fig 6H). Taken together, these *in vitro* gene expression data suggested that *Map3k8* ^*–/–*^ M2-MΦ’s had both elevated pro-fibrotic properties and reduced regulatory/inhibitory functions (*Arg1* and *Retnla* (Figs 4 and 5)).

### *Map3k8* regulates lipolysis for proficient alternative activation of Macrophages

**Fig 7:**
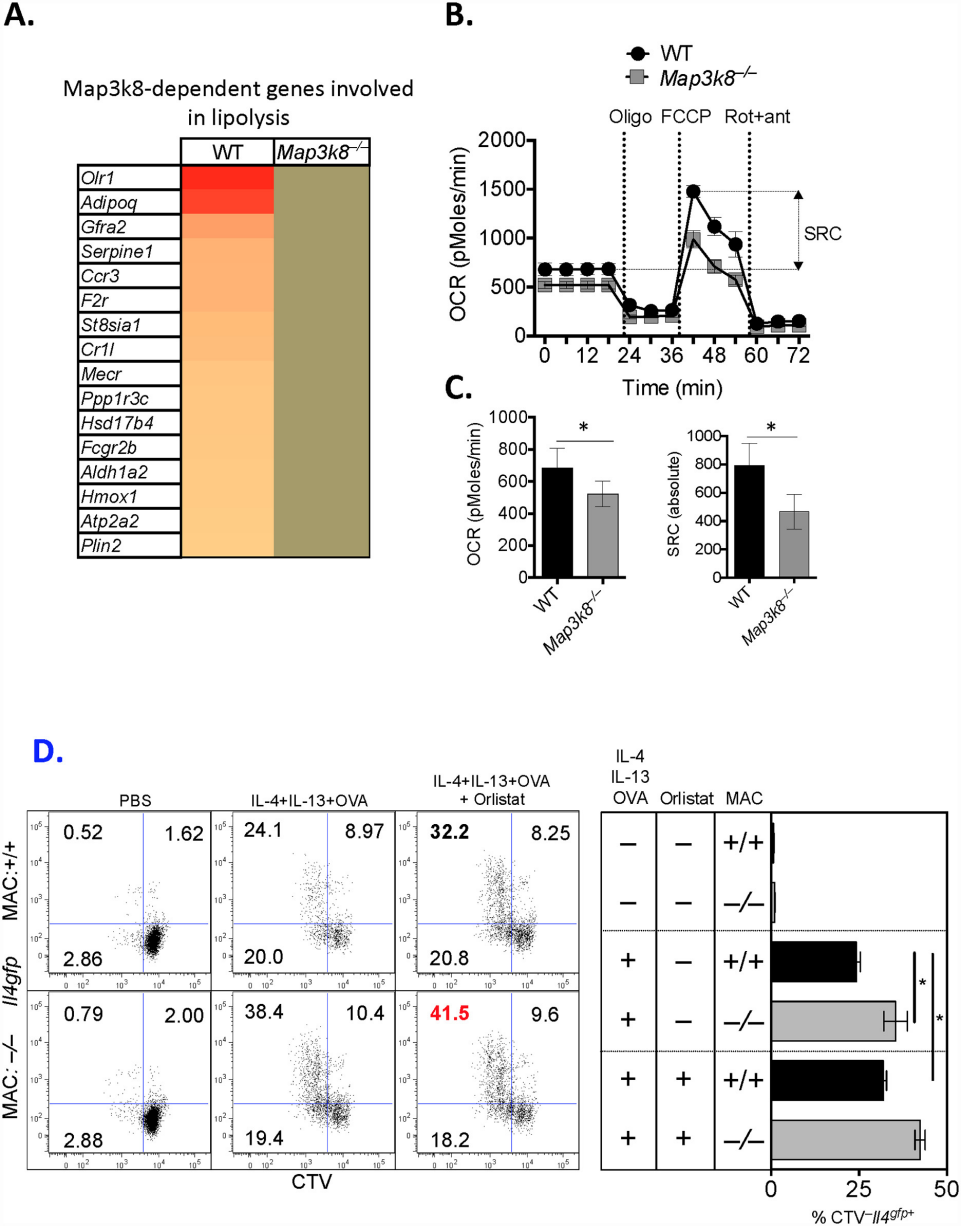
TPL-2 regulates lipolysis in M2 macrophages and regulation of T_H_2 cell differentiation and proliferation. Bone marrow-derived macrophages (BMDM) were stimulated with IL-4 and IL-13 for 24 hours. A) Analysis of genes involved in lipid metabolism was performed by Ingenuity pathways analysis (S1 Table) with *Map3k8*-dependent genes depicted in a heat map. B) After 24hrs of stimulation with IL-4 and IL-13 oxygen consumption rates (OCR) were determined in M2 macrophages using an XF-96 Extracellular Flux Analyzer (EFA) during sequential treatments with oligomycin, FCCP, and rotenone/antimycin. Spare respiratory capacity (SRC), the quantitative difference between maximal uncontrolled OCR, and the initial basal OCR, is depicted in the plot. C) Basal oxygen consumption rates (OCR) and spare respiratory capacity (SRC) in WT and *Map3k8* ^*–/–*^M2 macrophages. D) WT or Map3k8-deficent bone marrow-derived macrophages (BMDM) were generated from 3 individual mice and co-cultured with a pool of cell trace Violet (CTV)-labelled naïve OTII CD4^+^CD44^−^ *Il4* ^*gfp*–^T cells and stimulated with IL-4 and IL-13 for 3 days. Th2 cell differentiation (*Il4* ^*gfp*^ expression) and proliferation (CTV dilution) was determined by FACS after 3 days. In some wells BMDM were pre-treated with Orlistat for 6 hours and washed, prior to co-culture. Data is representative of 2–3 independent experiments with a minimum of 3 biological replicates per experiment. * p< 0.05 as assessed by two-tailed Mann-Whitney test.

Oxidative lipid metabolism is a metabolic programme recently reported to be essential for M2-MΦ activation [44]. It also has previously been reported that TPL-2, MEK 1/2 and ERK 1/2 [45–48] can regulate lipid metabolism in a variety of different cells. We therefore hypothesised that the compromised M2-MΦ activation of *Map3k8*-deficient MΦ’s was due to reduced lipid metabolism. To investigate this possibility, we analysed the expression of 220 genes involved in lipid metabolism from the transcriptional data obtained from WT and *Map3k8* ^*–/–*^ M2-MΦ ‘s (S1 Table) and identified 16 TPL-2-dependent genes involved in lipid metabolism that were up regulated in WT M2-MΦ’s but not in *Map3k8* ^*–/–*^ M2-MΦ’s (Fig 7A). These genes included the LDL receptor, *Olr1*, which is required for lipid uptake [49] and *Adipoq* encoding adiponectin, which promotes lipid oxidation [50] and the alternative activation of human MΦ’s [51]. In addition, *Aldh1a2* (also referred to as *Raldh2*) which catalyses the synthesis of the lipid metabolism-promoting metabolite, retinoic acid, from retinaldehyde [52] and the NFκB-regulated sialyltransferase, *St8sia1* [53], which catalyzes the transfer of sialic acid from CMP-sialic acid to GM3 to produce gangliosides, were reduced in *Map3k8* ^*–/–*^ M2-MΦ’s, compared to WT M2-MΦ’s. Several of these genes are downstream of TPL-2 (S5 Fig), supporting our hypothesis that TPL-2 regulates lipolysis in M2-MΦ’s. Together these changes in gene expression in *Map3k8*-deficient M2-MΦ’s were consistent with TPL-2 signalling regulating lipolysis.

To formally test whether lipid metabolism was compromised in *Map3k8* ^*–/–*^ M2-MΦ’s, we used extracellular flux analysis and measured the oxygen consumption rate (OCR) and spare respiratory capacity (SRC, the quantitative difference between maximal uncontrolled OCR, and the initial basal OCR, indicative of commitment to oxidative phosphorylation) in un-stimulated and IL-4/IL-13 mediated M2-MΦ’s. At baseline, un-stimulated WT and *Map3k8* ^*–/–*^MΦ’s had a similar OCR and SRC (S6 Fig). However, *Map3k8* ^*–/–*^ M2-MΦ’s had significantly reduced OCR and SRC (Fig 7B and 7C), indicating that TPL-2 is required for lipid metabolism in M2-MΦ’s, and providing a mechanistic explanation for reduced M2-MΦ’s in *Map3k8* ^*–/–*^mice.

*Map3k8* ^–/–^mice and *LysM* ^*Cre*^ *Map3k8* ^*fl/fl*^ mice, which had compromised M2-MΦ activation, had elevated Th2 cell responses. It has previously been reported that M2-MΦ’s can directly regulate T cell responses [13–15]. We therefore hypothesised that *Map3k8* ^*–/–*^ M2-MΦ’s would not regulate Th2 cell differentiation and proliferation as well as WT M2-MΦ’s. To test this hypothesis, we co-cultured WT or *Map3k8* ^*–/–*^BMMΦ’s with naïve cell trace violet (CTV)-labelled CD4^+^CD44^−^ *Il4* ^*gfp*–^OTII^+^ T cells in the presence of IL-4, IL-13 and OVA and determined the proliferation (CTV dilution) and differentiation (*Il4* ^*gfp*^ expression) of T cells. After 3 days, 24% of T cells had proliferated and differentiated when co-cultured with WT MΦ’s (Fig 7D, top row middle panel). However, co-culture of T cells with *Map3k8* ^*–/–*^MΦ’s led to significantly more Th2 cell differentiation and proliferation (~38%, Fig 7D, bottom row middle panel). Finally, to determine whether lipid metabolism contributed to MΦ-mediated regulation of Th2 cell proliferation and differentiation we pre-treated MΦ’s for 6 hours with Orlistat, an irreversible lipase inhibitor, prior to co-culture with T cells. Orlistat treated WT MΦ’s led to more Th2 cell proliferation and differentiation (~32%, Fig 7D, top row right panel), phenocopying *Map3k8* ^*–/–*^MΦ’s and indicating that lipid metabolism in IL-4/IL-13 activated MΦ’s was required for optimal MΦ-mediated control of Th2 cell proliferation, as previously reported [44]. Of note, Orlistat treated *Map3k8* ^*–/–*^MΦ’s only led to a small increase in Th2 cell proliferation, suggesting that lipid metabolism was already at a minimum in *Map3k8* ^*–/–*^MΦ’s.

Taken together this study has demonstrated that TPL-2 is a critical regulator of immune-mediated pathology and fibrosis following *S*. *mansoni* infection, functioning as an important metabolic regulator in M2-MΦ activation.

## Discussion

Liver fibrosis and cirrhosis, which is responsible for over 1.5 million fatalities per year [54], can develop following a variety of infectious insults, including chronic infection [4]. Diseases characterized by persistent T_H_2 cytokine responses, such as chronic helminth infections, are associated with the development of significant tissue pathology and fibrotic scarring. Although the molecular pathogenesis of fibrotic diseases are slowly emerging [4], there are very few novel therapeutic candidates progressing through clinical trials [55], highlighting a significant unmet medical need.

Two distinct inflammatory axes contribute to inflammation-driven fibrosis; type-1/ T_H_17 mediated inflammation [30] and type-2 driven fibrosis [56]. In this study we established that the Map3 kinase, TPL-2, is an important negative regulator of chronic type-2 inflammation-driven fibrosis following schistosome infection. These data are in contrast to a previous study testing the role of TPL-2 in three models of pro-inflammatory type-1/17-associated fibrosis (carbon tetrachloride-, methionine-choline-deficient diet- and bile duct ligation-induced fibrosis)[57, 58]. In two of these three models *Map3k8* ^*–/–*^mice had significantly reduced fibrosis [32]. These seemingly contrasting results most likely reflect the different inflammatory events contributing to the fibrogenic response. For example, it has been widely reported that TPL-2 is required for pro-inflammatory type-1/17-associated inflammation and immunity [17–29]. It therefore stands to reason that TPL-2 would be required for pro-inflammatory type-1/17-associated fibrosis, as reported by Perugorria and colleagues [32]. In contrast, TPL-2 appears to function as a negative regulator of type-2 inflammation in the lung and liver (Fig 1, S2 Fig), with increased acute [35] and chronic type-2 inflammation in *Map3k8* ^–/–^mice, as presented here. In this context, the exacerbated type-2 inflammatory response in *Map3k8* ^–/–^mice resulted in increased fibrosis. If these animal models reflect human disease, focused strategies targeting TPL-2 would benefit from identifying a prognostic biomarker and treating patients with type-1/17-associated fibrosis, rather than patients with type-2-associated fibrosis.

Inflammation-driven fibrosis involves a co-ordinated and often dysregulated wound healing response involving a variety of migratory leukocytes activating local stroma. TPL-2 is expressed in both leukocytes and local stroma and therefore identifying where TPL-2 was regulating the fibrogenic process was essential for us to identify how TPL-2 regulated fibrosis. It has been previously suggested that increased acute T_H_2 responses in *Map3k8* ^–/–^mice was due to a T cell-intrinsic role for TPL-2 [35], however this was not tested *in vivo*. Similarly, increased intestinal inflammation and tumorigenesis in *Map3k8* ^–/–^mice was attributed to a reduced frequency of Foxp3^+^ T_REG_ cells, [36], however again this was not specifically tested *in vivo*. Using *Map3k8* ^–/–^ *Il4* ^*gfp*^ *Foxp3* ^*rfp*^ mice and re-stimulated local lymph nodes we identified that TPL-2 negatively regulated the differentiation, expansion and/or recruitment of T_H_2 cells, however this was not due to a T cell-intrinsic role for TPL-2, as mice with a T cell-intrinsic deletion of *Map3k8* mounted similar T_H_2 responses as WT mice. Furthermore, exacerbated hepatic fibrosis observed in *Map3k8* ^–/–^mice was not observed in mice with a T cell-specific deletion of *Map3k8*, indicating that T cell-intrinsic TPL-2 had no impact on type-2–mediated inflammation or fibrosis in these systems.

TPL-2 has been extensively studied in TLR-mediated classical macrophage activation (CA, M1-MΦ) [20]. However, it was unclear whether TPL-2 contributes to M2-MΦ differentiation. M2-MΦ’s are central regulators of inflammation, wound-healing and fibrosis following schistosome infection [12]. Specifically, *Arginase* (*Arg-1*) in M2-MΦ’s catalyses the cleavage of arginine to ornithine and urea, depleting extracellular arginine and depriving local leukocytes of this essential amino acid. Consequently, M2-MΦ’s limit T cell proliferation, by starvation, in an *Arg-1*-dependent manner [14]. Similarly, production of *Retnla* by M2-MΦ’s can suppress T cell responses directly [13, 15]; highlighting two mechanisms by which M2-MΦ’s regulate T_H_ cell responses. The reduced expression of these immunoregulatory molecules (*Arg1* and *Retnla*) in *Map3k8* ^*–/–*^ M2-MΦ’s may therefore explain the elevated T_H_2 cell inflammation observed in *Map3k8* ^*–/–*^mice. Indeed, *Map3k8* ^*–/–*^ M2-MΦ’s did not control T_H_2 cell differentiation or proliferation (Fig 7D).

M2-MΦ’s also supply proline for collagen synthesis and produce a variety of pro-fibrotic factors to promote wound healing, collagen deposition and if dysregulated, fibrotic scarring [59]. We observed increased expression of pro-fibrotic genes (*Col1a1*, *Col3a1*, *Col5a2* and *Ctgf*) in *Map3k8* ^*–/–*^ M2-MΦ’s, which may explain the elevated fibrosis observed in *Map3k8* ^*–/–*^mice. The mechanism of TPL-2-mediated regulation of pro-fibrotic mediators is not completely understood. It was recently reported that TPL-2 regulates hepatocyte growth factor (HGF) production in fibroblasts [60], in part, by reducing sensitivity to TGFβ. In IL-13-dependent fibrosis, TGFβ can inhibit some pro-fibrotic pathways [33]. If *Map3k8*-deficient M2-MΦ’s also have decreased sensitivity to TGFβ, this may explain the increased expression of several pro-fibrotic mediators in *Map3k8*-deficient M2-MΦ’s, however this requires further study. Together, these two observations provide some explanations for the increased T_H_2 cell responses and exacerbated hepatic and pulmonary fibrosis observed in *Map3k8* ^–/–^mice. Indeed, specific deletion of *Map3k8* in LysM-expressing cells phenocopied *Map3k8* ^–/–^mice, with increased T_H_2 inflammation and fibrosis following *S*. *mansoni* infection or *S*. *mansoni* egg injection, compared to WT mice. The *LysM* ^*Cre*^ system appears to efficiently delete floxed alleles in tissue-resident MΦ’s, but not so well in newly recruited myeloid cells [61, 62]. The exacerbated immunopathology observed in *LysM* ^*Cre*^ *Map3k8* ^fl/fl^ mice suggests that *Map3k8* may have an important function in tissue-resident MΦ’s, rather than newly recruited myeloid cells that may not have efficiently deleted *Map3k8*.

Exactly how TPL-2 regulates IL-4R-driven M2-MΦ activation is unclear. TPL-2, and downstream kinases, MEK 1/2 and ERK 1/2 are required for lipid metabolism [45–48], a metabolic programme recently identified to be essential for M2-MΦ [44]. Joining these two independent observations, we confirmed that TPL-2 was an essential regulator of lipid metabolism in M2-MΦ’s, with extracellular flux assays identifying reduced oxygen consumption and reduced spare respiratory capacity, compared to WT M2-MΦ’s. The decreased lipid metabolism in *Map3k8* ^–/–^ M2-MΦ’s provides an explanation for the decreased expression of immunoregulatory molecules [44] in *Map3k8* ^–/–^ M2-MΦ’s. In addition, *Map3k8* ^–/–^ M2-MΦ’s had reduced expression of several genes encoding enzymes involved in lipid uptake, lipid metabolism and synthesis (Fig 6), providing an explanation as to how lipid metabolism may be compromised in *Map3k8* ^–/–^ M2-MΦ’s. Thus, we speculate that following IL-4R-signalling and STAT-6-mediated activation of downstream gene products, TPL-2 is required for the necessary metabolic re-programming [44] for optimal M2-MΦ activation.

A paralleled increase in pro-fibrotic factors in *Map3k8* ^–/–^ M2-MΦ’s identified a novel TPL-2-regulated pro-fibrotic axis in M2-MΦ’s. Whether this was also due to compromised metabolic programme in *Map3k8* ^–/–^ M2-MΦ’s is currently unclear. *In vivo*, the increased T_H_2-mediated inflammation, as a result of compromised immune regulation by *Map3k8* ^–/–^ M2-MΦ’s, may further exacerbate pro-fibrotic pathways, providing a severely dysregulated microenvironment. In summary, this study has identified that TPL-2 is an important metabolic regulator in M2-MΦ’s *in vitro* and that myeloid cell intrinsic TPL-2 critically controlled chronic type-2-mediated inflammation and fibrosis *in vivo*.

## Materials and Methods

### Animals

All mice were bred and maintained under specific pathogen-free conditions at The Francis Crick Institute. Strains used included: WT C57BL/6, *Map3k8* ^*–/–*^[20], *OTII* [63], *Il4* ^*gfp*^ [64], Foxp3^rfp^[65], *Cd4* ^*Cre*^ *Map3k8* ^*flfl*^ *R26* ^*eYFP*^ (B6.*Cd4* ^*Cre*^ [66] *crossed with Map3k8* ^*flfl*^ *and* B6.*R26* ^*eYFP*^), *LysM* ^*Cre*^ *Map3k8* ^*flfl*^ *R26* ^*eYFP*^ (B6.*LysM* ^*Cre*^ [67] *crossed with Map3k8* ^*flfl*^ *and* B6.*R26* ^*eYFP*^).

### *S*. *mansoni* infection and egg-induced pulmonary inflammation

Mice were infected percutaneously via the tail with 50 cercariae of a Puerto Rican strain of *S*. *mansoni* (NMRI) obtained from *Biomphalaria glabrata* snails, kindly provided by Dr. Quentin Bickle, LSHTM. Infection intensity was determined following perfusion and granuloma size was determined from 10–20 individual granulomas per tissue sample, measured using Image J. Tissue pathology was analysed following Masson’s trichrome (Collagen, Blue; Nuclei, black/dark blue; Muscle, cytoplasm, Red) staining of 5μm sections from paraffin embedded samples. Intestinal pathology was determined using a comprehensive scoring system taking into account the level of infiltration, disruption and severity of the intestinal architecture [68]. Hydroxyproline content was quantified in liver tissue using a hydroxyproline assay kit according to the manufacturers recommendations (Cambridge Biosciences, UK). Tissue eggs were quantified by digesting a known weight of liver tissue with collagenase and liberase and isolating eggs on a discontinuous percoll gradient, as previously described [69]. For intravenous delivery of *S*. *mansoni* eggs, eggs were washed extensively in PBS, with 5000k delivered in 200μl of sterile PBS.

### Bone marrow derived macrophage culture

Bone marrow cells were plated to a density of 5 x 10^6^ cells per 90-mm bacterial Petri dish (Sterilin) in 10ml of DMEM/F-12 Dulbecco’s Modified Eagle Medium/Nutrient Mixture F-12 with GlutaMAX supplement (Gibco) supplemented with 10% FBS, antibiotics, 20% L-cell conditioned medium, L-Glutamine (1%), HEPES (1%), Sodium Pyruvate (1%) and β-mercaptoethanol (1%). After 4 days of culture, 10ml of additional medium was added and cells were cultured for a further 3 days. Non-adherent cells were washed away and the adherent cells were collected in 5ml PBS with 5% FBS and 2.5mM EDTA. For experiments the cells were re-plated in medium with 1% FBS without L-cell supplement and were incubated overnight before stimulation. Cells were stimulated with IL-4 and IL-13 (20ng/ml) (R&D systems) or LPS (100ng/ml) (Alexis Biochemicals). In some experiments, BMDM were generated from three individual mice and co-cultured with a pool of naïve OTII CD4^+^CD44^−^ *Il4* ^*gfp*–^T cells during stimulation with OVA peptide _(323–339)_ (Invivogen). In some of these co-culture experiments, BMDM were pre-treated for 6 hours with Orlistat (100μM; Cayman), prior to co-culture.

### Isolation of hepatic macrophages

Liver tissue was perfused and the organ was collected in gentleMACS columns (Miltenyi Biotec) in incomplete RPMI 1640 (Gibco). The tissues were dissociated in incomplete RPMI 1640 with Liberase TL (0.5mg/ml) (Roche), Collagenase (4μg/ml) (Roche) and DNAse (7.5μg/ml) using the gentleMACS dissociator (Miltenyi Biotec). The partly digested tissue fragments were incubated at 37°C for 45 min, following which the tissues were completely dissociated. The cellular fraction was run through a 100μm filter and the cells were centrifuged at 50g for 3 min, to pellet the non-parenchymal cells and the supernatant fraction was centrifuged at 320g for 5min. The cellular fractions from both steps were pooled and collected in 1X HBSS Hanks balanced salt solution and mixed with OptiPrep (Sigma) to get a 17% w/v solution and overlayed with 1X GBSS Gey’s balanced salt solution. The samples were centrifuged at 400g for 15min at room temperature with no brakes and the enriched layer of cells were collected from the interface of the GBSS and the 17% solution. The cells were stained and FACS sorted as Live/ CD45^+^/CD3^−^/CD19^−^/NK1.1^−^/Ly6G^−^/SiglecF^−^/CD11b^+^/F4/80^+^.

### Metabolism assays

Day 7 bone marrow-derived monocytes (BMDM) were cultured at a density of 5x10^5^ cells in an XF24 plate (Seahorse Bioscience) over night. On day 8 cells were stimulated with 20ng/ml of recombinant IL-4 and IL-13 (R&D systems). On day 9 media was replaced with XF base medium (Seahorse Bioscience) supplemented with 100x Glutamax (Gibco), 100X Sodium Pyruvate (SIGMA) and 25mM glucose and the plate incubated for 10–30 minutes in a non-CO_2_ incubator at 37°C. For analysis of basal oxygen consumption rate (OCR) and extracellular acidification rate (ECAR), cells were analysed with XF-24 Extracellular Flux Analyzer (Seahorse Bioscience) under standard Seahorse running protocol. The Seahorse cartridge was loaded at a final concentration of 10μM Oligomycin (SIGMA O4876-5mg), 15μM FCCP (triflourocarbonylcyanide phenylhydrazone, SIGMA C2920-10mg), 1μM Rotenone (SIGMA R8875) plus 10μM Antimycin A (A8674-25mg) in ports A, B and C respectively. The bioenergetics profile consisted of basal OCR measurements in the absence of drugs and OCR/ECAR following the injection of drugs. All OCR/ECAR/SRC analyses were obtained from 5 replicates in 3 independent repeats.

#### LC-MS for arginase assay

At day 7, non-adherent cells were washed away and the adherent cells were collected in 5ml PBS with 5% FBS and 2.5mM EDTA. Cells were re-plated in medium with 1% FBS without L-cell supplement in a 6-well plate. Cells were kept for 12–18 hours, then stimulated with IL-4 and IL-13 (20ng/ml) (R&D systems) for 24hrs. Cells were washed twice with PBS, metabolites were extracted with ice cold mixture of acetonitrile/methanol/water (2:2:1, v/v/v). Plates were kept on dry ice for 5–10 min. Cells were collected, vortexed vigorously then spun for 20 min at 16,000g at 4°C. The supernatant was stored at -80°C until analysis. Residual protein content in the cell lysates was determined with Bicinchoninic acid method (BCA assay, Pierce). Prior to the LC-MS analysis, 100μL of the cell lysates were acidified with an equal volume of acetonitrile containing 0.2% acetic acid. The mixture was spun for 10 minutes at 16,000g, at 4°C. 2μL of the acidified mixture were used for the LC-MS analysis. Chromatography was performed on Agilent 1200 LC system, including a solvent degasser, binary pump and temperature-controlled auto-sampler. Samples were inject to a Cogent Diamond Hydride Type C silica column (150mm×2.1mm i.d, 4μm particle size and 100A pore size) and eluted with flow rate of 0.4 mL/min. The gradient employed is based on the number 3, according to [70]. Metabolites were detected using an Agilent Accurate Mass 6230 TOF apparatus, as previously described [71]. Detected m/z 175.11895 at 16.8 min and 133.0972 at 16.6 min were identified as arginine and ornithine, respectively, on the basis of unique accurate mass-retention time identifiers and spectral data. The reported abundances were normalized to the residual protein content in each corresponding sample.

### T_H_2 polarization and re-stimulation of lymph node cells ex-vivo

Naive CD4^+^ T cells (CD4^+^TCRβ^+^CD44^−^CD25^−^PI^−^) were FACS-purified from spleens of WT or *Map3k8* ^*–/–*^mice. Naive T cells were cultured for 6 days *in vitro* with 10ng/mL IL-4 (R&D), 5ng/mL IL-2 (R&D), 10 μg/mL anti-IFNγ (XMG1.2, BioXcell), and CD3 (0.1–4.0μg) (145-2C11, BioXcell) and CD28 (10μg/ml) (37.51, BioXcell) in complete IMDM (cIMDM, 10% fetal calf serum (FCS),100 U/mL Penicillin and 100 μg/mL Streptomycin (Gibco), 8mM L-glutamine (Gibco), and 0.05mM 2-mercaptoethanol (Gibco)). For re-stimulation of lymph node cells ex-vivo, lymph nodes were disrupted into a single cell suspension with 2x10^5^ cells cultured in a 96-well, round bottom plate with 10μg/ml of anti-CD3 (145-2C11, BioXcell). Supernatants were harvested after 3 days for analysis by ELISA.

### qRT-PCR, ELISA, western blotting and serum LP

#### qRT-PCR

Tissue samples were frozen in RNAlater (Sigma) and homogenized in QIAzol (Qiagen). Cell pellets were lysed in Buffer RLT (Qiagen). Total RNA was isolated as per manufacturer’s protocol. 100ng-1μg of total RNA was reverse transcribed using the QuantiTect Reverse Transcription Kit (Qiagen). cDNA produced was used for real time quantitative PCR with Power SyBrGreen (Applied Biosystems). The expression levels of different genes were normalised to hypoxanthine-guanine phosphoribosyl transferase (HPRT) expression and expressed as fold change relative to naïve or PBS-treated WT samples.

#### ELISA

IL-5, IL-13, IL-10, IFNγ and IL-17 were measured using DuoSet ELISA kits, according to the manufacturer’s instructions (R&D systems—Biotechne).

#### Western blotting

For immunoblotting, cell lysates were normalized to equal total protein content and resolved on 10% Criterion TGX Gels (Biorad). Separated proteins were transferred onto Trans-Blot Turbo PVDF transfer (Biorad) membranes (Immobilon). Specific bound antibodies were visualized by chemiluminescence (Merck Millipore). Endotoxin levels in serum samples were measured using an LAL chromogenic assay, according to manufacturer’s recommendations (Pierce, Thermo fisher).

### Cell sorting and flow cytometry

Cell sorting was performed using a FACS Aria II (BD Biosciences) cell sorter. For sorting, cell suspensions were stained for 20 minutes with antibodies in PBS with 2% fetal calf serum (FCS) and then diluted in phenol-red free IMDM (Gibco) (with 1% FCS, 2mM EDTA (Invitrogen), 100U/mL Penicillin and 100μg/mL Streptomycin (Gibco), 8mM L-glutamine (Gibco), and 0.05mM 2-mercaptoethanol (Gibco)). Propidium iodide (PI) or LIVE/DEAD fixable blue dead cell stain (Life Technologies) was used to determine cell viability. Cells were stained for surface antigens by incubation with antibodies in PBS with 2% FCS (20 minutes at 4°C). Intracellular cytokine staining was performed following 6 hours re-stimulation with 50ng/mL phorbol 12-myristate 13-acetate (PMA, Promega) and 1μg/mL ionomycin (Sigma) and BD Golgi Stop and BD Golgi Plug (diluted 1:1000, BD Biosciences). After staining for surface antigens, cells were fixed and permeabilized (Fixation/Permeabilization diluent; eBioscience), prior to incubation with cytokine antibodies in Permeabilization buffer (eBioscience) for 20 min at 4°C. Cells were analyzed using a BD LSRII flow cytometer (BD Biosciences) and data processed using FlowJo software (Version X 10.0.7r2, Treestar Inc). Antibodies used were purchased with eBioscience, Biolegend or BD Pharmingen. They included: CD45 (30-F11), CD3 (17A2), CD4 (RM4-5, GK1.5), CD11b (M1/70), CD19 (6D5, eBio1D3), CD25 (PC61), CD44 (IM7), NK1.1 (PK136), Ly6G (1A8), SiglecF (E50-2440), and F4/80 (BM8), TCR β chain (H57-597). Staining was performed in the presence of FcR Blocking Reagent (Miltenyi Biotec).

### RNA, qRT-PCR and microarray

RNA was isolated from tissues and cells using RNAeasy mini spin columns according to manufacturers’ instructions (Qiagen). cDNA was generated from 5ng of total RNA using WT-Ovation Pico system (version 1) RNA Amplification System followed by double stranded cDNA synthesis using WT-Ovation Exon Module. cDNA quality was determined using an Agilent BioAnalyzer and through hybridization performance on Affymetrix GeneChip mouse Genome 430A 2.0 microarray (Affymetrix) by the Systems Biology Unit at The Francis Crick Institute. Microarray data were quantile-normalized and analysed using GeneSpring software (Agilent). Differentially expressed genes were determined using ANOVA and t-tests. Genes with false discovery rate corrected p-values less than 0.1 and fold change values ≥1.5 were considered significant, and as indicated in figure legends. Three biological replicates of each subset were used. Pathways analysis was performed using Ingenuity Pathways Analysis (IPA, Ingenuity Systems, www.ingenuity.com).

### Statistical analysis

Data sets were compared by Mann Whitney test using GraphPad Prism (V.5.0). Differences were considered significant at *p ≤ 0.05 using one or two-tailed tests.

### Ethics statement

All animal experiments were carried out following UK Home Office regulations (project license 80/2506) and were approved by The Francis Crick Institute Ethical Review Panel.

## Supporting Information

S1 Fig TPL-2 regulated parasitology and pathology. WT and *Map3k8* ^*–/–*^mice were infected percutenously with 50 *S*. *mansoni* cercariae and analysed at 8 weeks post-infection. A) *S*. *mansoni* eggs were quantified in the liver tissue as previously described [69]. B) Endotoxin levels (LPS) in serum was determined using an LAL assay kit at necropsy. C) Expression of *Il13*, *Il1b*, *Tgfb*, *Il17a*, *Ifny*, *Tnfa* and *Il6* was determined from RNA extracted from liver tissue. Data is expressed relative to HPRT and presented as a fold-change relative to genotype-controlled naïve mice.
(TIFF)

S2 Fig *Map3k8* ^–/–^mice develop increased *S*. *mansoni* egg-induced pulmonary fibrosis. WT or *Map3k8* ^*–/–*^mice were given 5000 *S*. *mansoni* eggs intravenously before necropsy at day 21. A) Lung tissue was fixed and embedded in paraffin before sectioning and staining with Masson’s trichrome. B) Hydroxyproline was quantified in liver tissue from naïve and *S*. *mansoni* egg treated mice. C) Frequency of T_REG_ (CD4^+^ *Foxp3* ^*RFP*+^) and T_H_2 (CD4^+^ *Il4* ^*GFP*+^) cells in the thoracic lymph nodes (top row) and lung (bottom row) were determined by FACS on day 21. D) Expression of *Il13*, *Col6* and Mmp12 was determined in RNA extracted from lung tissue. Data is expressed relative to HPRT and presented as a fold-change relative to genotype-controlled naïve mice. All experiments are representative of 2 independent experiments with 5 mice/genotype. * p< 0.05 as assessed by two-tailed Mann-Whitney test.
(TIFF)

S3 Fig Myeloid cell (*LysM* ^+^) expression of Map3k8 regulates T_H_2-mediated immunopathology and fibrosis following *S*. *mansoni* infection. *LysM* ^*Cre*^ *Map3k8* ^*+/+*^ and *LysM* ^*Cre*^ *Map3k8* ^*fl/fl*^ mice were infected percutenously with 50 *S*. *mansoni* cercariae and analysed at 8 weeks post-infection. A) Detection of TPL-2 protein in macrophages (Live/Dead^−^CD45^+^F4/80^+^LysM^Cre^R26e^YFP+^) from *LysM* ^*Cre*^ *Map3k8* ^*+/+*^ and *LysM* ^*Cre*^ *Map3k8* ^*fl/fl*^ mice. B) Endotoxin levels (LPS) in serum was determined using an LAL assay kit at necropsy. C) Expression of *Il13*, *Il1b*, *Tgfb*, *Il17a*, *Ifny*, *Tnfa* and *Il6* was determined from RNA extracted from liver tissue. Data is expressed relative to HPRT and presented as a fold-change relative to genotype-controlled naïve mice.
(TIFF)

S4 Fig TPL-2 regulated macrophage activation. A) WT and *Map3k8* ^*–/–*^bone marrow-derived macrophages (BMDM) were stimulated with IL-4 and IL-13 for 24 hours with cell lysates used for arginine metabolism profiling using LC-MS. B) WT and *Map3k8* ^*–/–*^bone marrow-derived macrophages (BMDM) were stimulated with IL-4 and IL-13 for 24 hours in the presence of a specific TPL-2 inhibitor, C34. Cells were harvested, RNA extracted and *Retnla* expression was determined by qRT-PCR and expressed relative to un-stimulated genotype control cells.
(TIFF)

S5 Fig TPL-2 regulated lipid metabolism pathways in M2 macrophages. Ingenuity pathways analysis of lipid metabolism pathways (S1 Table) from bone marrow-derived macrophages (BMDM) stimulated with IL-4 and IL-13 for 24 hours, as in Figs 5 and 6. Elevated genes involved in lipid metabolism in WT, but not *Map3k8* ^–/–^macrophages, are displayed, with their relationship with *Map3k8* highlighted via intermediate genes.
(TIFF)

S6 Fig Lipolysis in un-stimulated WT and *Map3k8* ^*–/–*^macrophages. Bone marrow-derived macrophages (BMDM) were generated and left un-stimulated for 24 hours. A) Oxygen consumption rates (OCR) were determined using an XF-96 Extracellular Flux Analyzer (EFA) during sequential treatments with oligomycin, FCCP, and rotenone/antimycin. Spare respiratory capacity (SRC), the quantitative difference between maximal uncontrolled OCR, and the initial basal OCR, is depicted in the plot. B) Basal oxygen consumption rates (OCR) and (C) spare respiratory capacity (SRC) in WT and *Map3k8* ^*–/–*^macrophages. Data is representative of 3 independent experiments.
(TIFF)

S1 Table TPL-2 regulates lipolysis in M2 macrophages. Bone marrow-derived macrophages (BMDM) were stimulated with IL-4 and IL-13 for 24 hours, as in Fig 4. Analysis of genes involved in lipid metabolism was performed by Ingenuity pathways analysis. Gene expression is indicated, relative to un-stimulated macrophages. Highlighted genes are either absent in *Map3k8* ^–/–^M2 macrophages (Map3k8-dependent) or absent in both WT and *Map3k8* ^–/–^M2 macrophages.
(PDF)
