# Supplementary figures and images for: TPL-2 Regulates Macrophage Lipid Metabolism and M2 Differentiation to Control TH2-Mediated Immunopathology
Source: PLoS Pathog. 2016 Aug 3;12(8):e1005783. doi: 10.1371/journal.ppat.1005783 (PMC4972396; doi:10.1371/journal.ppat.1005783)

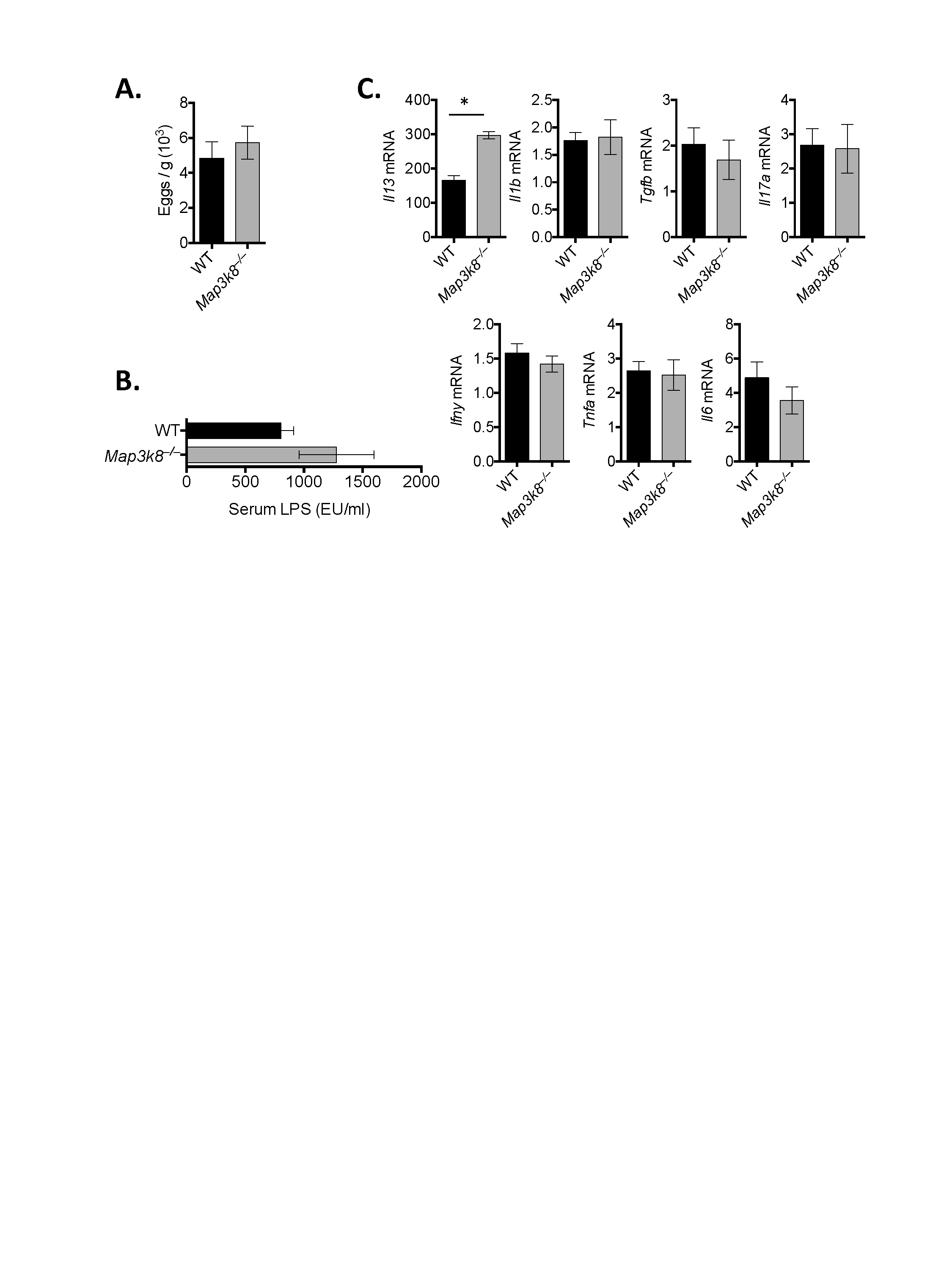

Supplement: S1 Fig — WT and Map3k8 –/–mice were infected percutenously with 50 S. mansoni cercariae and analysed at 8 weeks post-infection. A) S. mansoni eggs were quantified in the liver tissue as previously described [69]. B) Endotoxin levels (LPS) in serum was determined using an LAL assay kit at necropsy. C) Expression of Il13, Il1b, Tgfb, Il17a, Ifny, Tnfa and Il6 was determined from RNA extracted from liver tissue. Data is expressed relative to HPRT and presented as a fold-change relative to genotype-controlled naïve mice. (TIFF) [file ppat.1005783.s001.tiff]

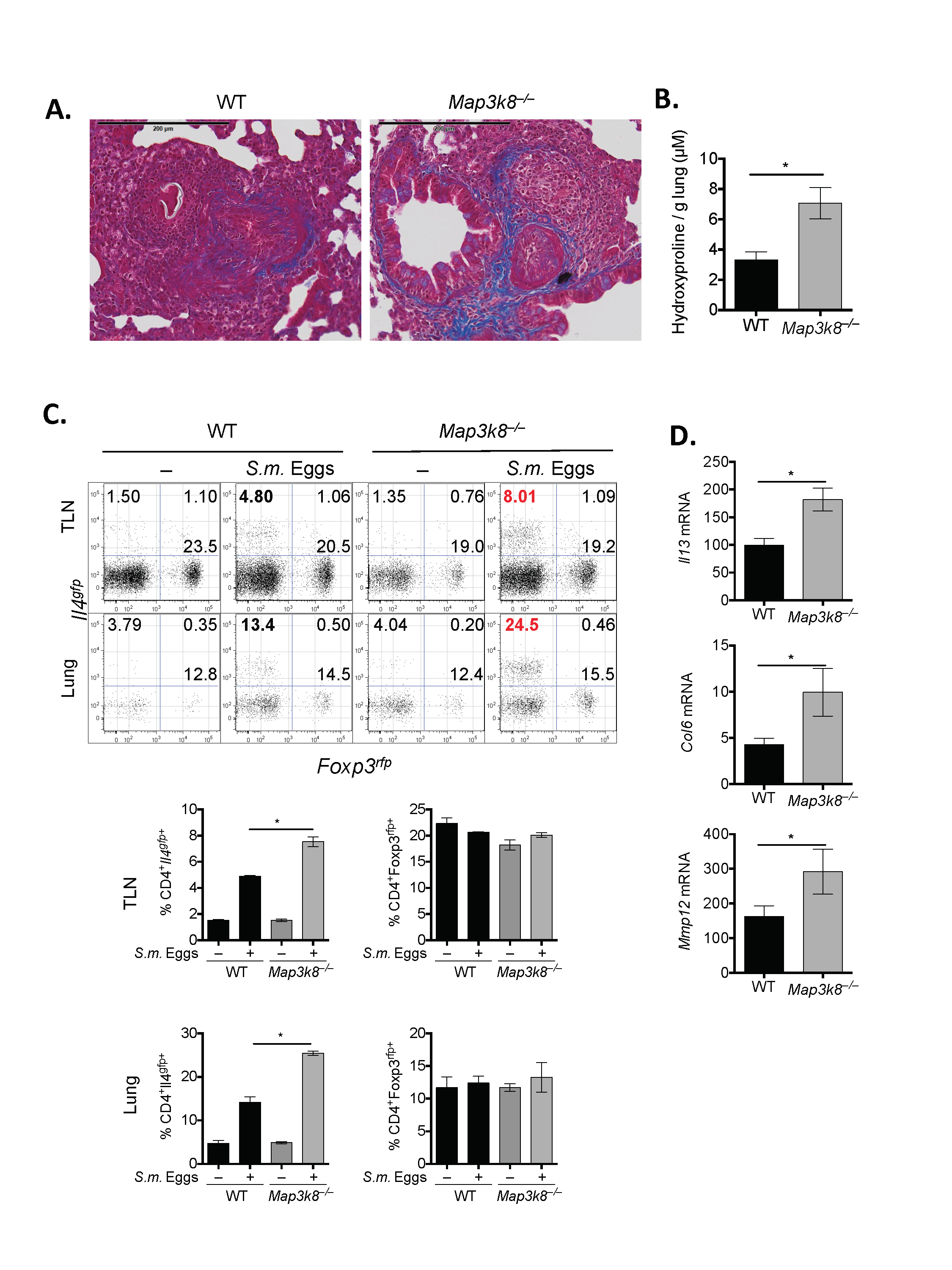

Supplement: S2 Fig — WT or Map3k8 –/–mice were given 5000 S. mansoni eggs intravenously before necropsy at day 21. A) Lung tissue was fixed and embedded in paraffin before sectioning and staining with Masson’s trichrome. B) Hydroxyproline was quantified in liver tissue from naïve and S. mansoni egg treated mice. C) Frequency of TREG (CD4+ Foxp3 RFP+) and TH2 (CD4+ Il4 GFP+) cells in the thoracic lymph nodes (top row) and lung (bottom row) were determined by FACS on day 21. D) Expression of Il13, Col6 and Mmp12 was determined in RNA extracted from lung tissue. Data is expressed relative to HPRT and presented as a fold-change relative to genotype-controlled naïve mice. All experiments are representative of 2 independent experiments with 5 mice/genotype. * p< 0.05 as assessed by two-tailed Mann-Whitney test. (TIFF) [file ppat.1005783.s002.tiff]

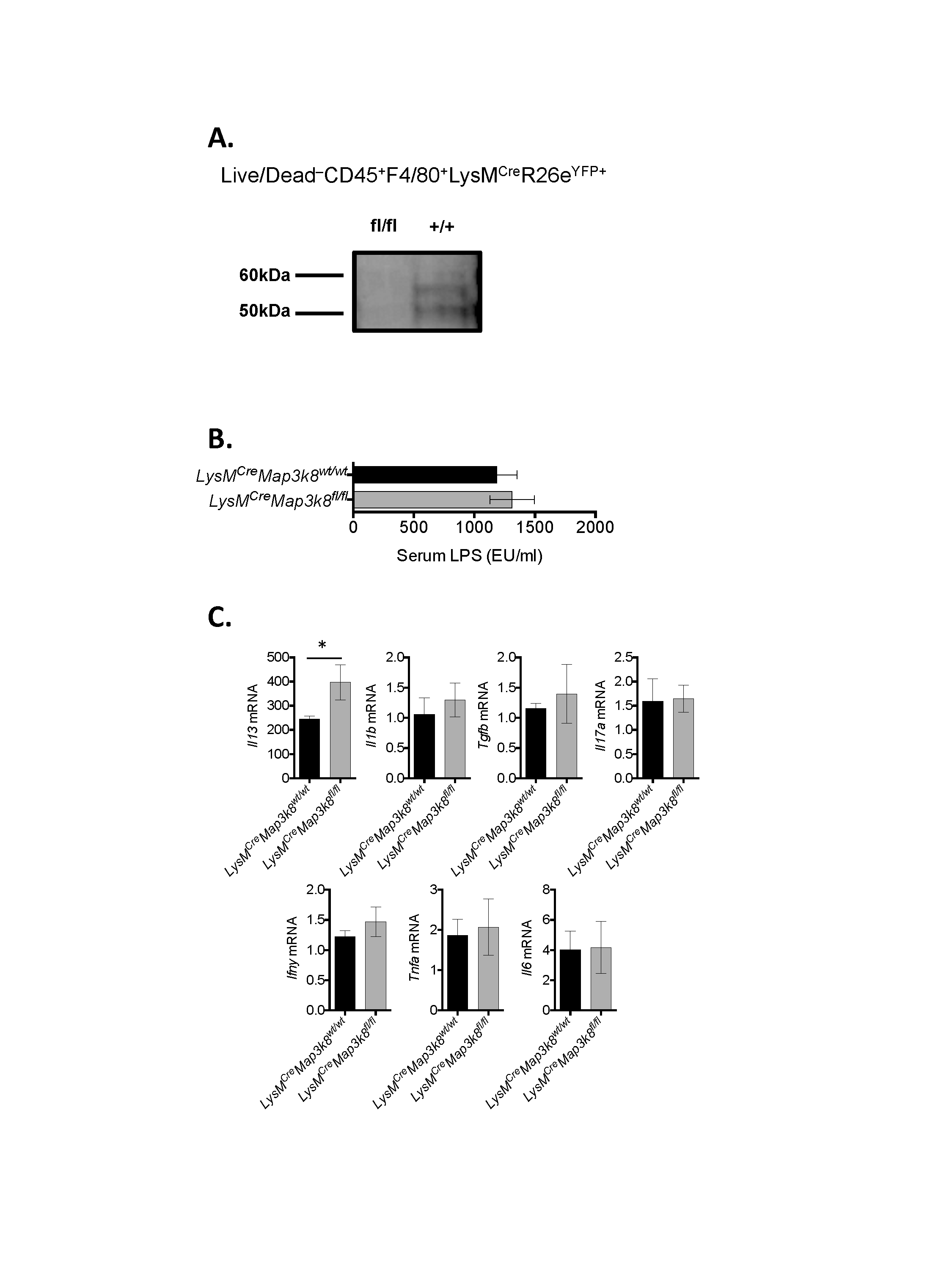

Supplement: S3 Fig — LysM Cre Map3k8 +/+ and LysM Cre Map3k8 fl/fl mice were infected percutenously with 50 S. mansoni cercariae and analysed at 8 weeks post-infection. A) Detection of TPL-2 protein in macrophages (Live/Dead−CD45+F4/80+LysMCreR26eYFP+) from LysM Cre Map3k8 +/+ and LysM Cre Map3k8 fl/fl mice. B) Endotoxin levels (LPS) in serum was determined using an LAL assay kit at necropsy. C) Expression of Il13, Il1b, Tgfb, Il17a, Ifny, Tnfa and Il6 was determined from RNA extracted from liver tissue. Data is expressed relative to HPRT and presented as a fold-change relative to genotype-controlled naïve mice. (TIFF) [file ppat.1005783.s003.tiff]

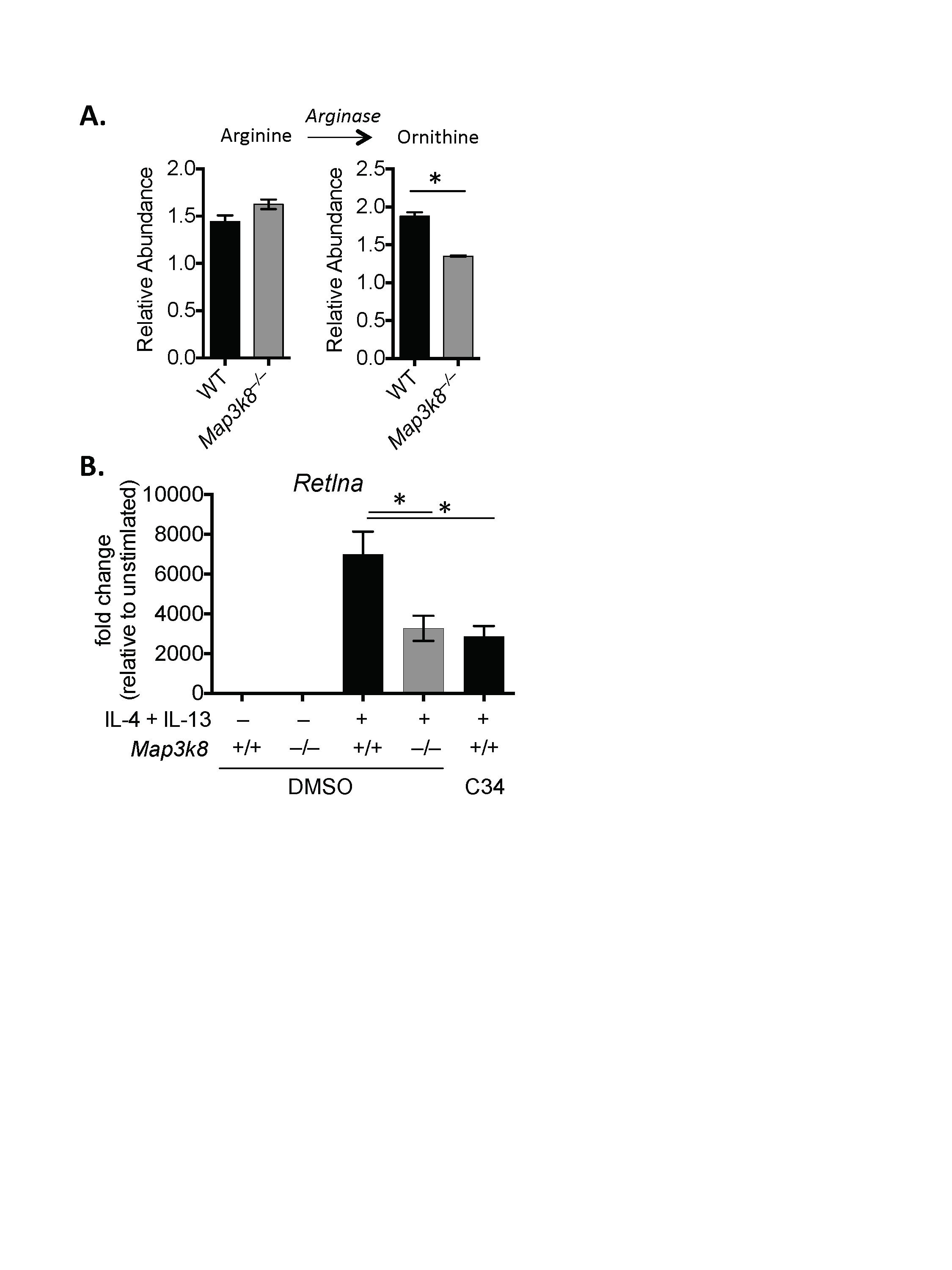

Supplement: S4 Fig — A) WT and Map3k8 –/–bone marrow-derived macrophages (BMDM) were stimulated with IL-4 and IL-13 for 24 hours with cell lysates used for arginine metabolism profiling using LC-MS. B) WT and Map3k8 –/–bone marrow-derived macrophages (BMDM) were stimulated with IL-4 and IL-13 for 24 hours in the presence of a specific TPL-2 inhibitor, C34. Cells were harvested, RNA extracted and Retnla expression was determined by qRT-PCR and expressed relative to un-stimulated genotype control cells. (TIFF) [file ppat.1005783.s004.tiff]

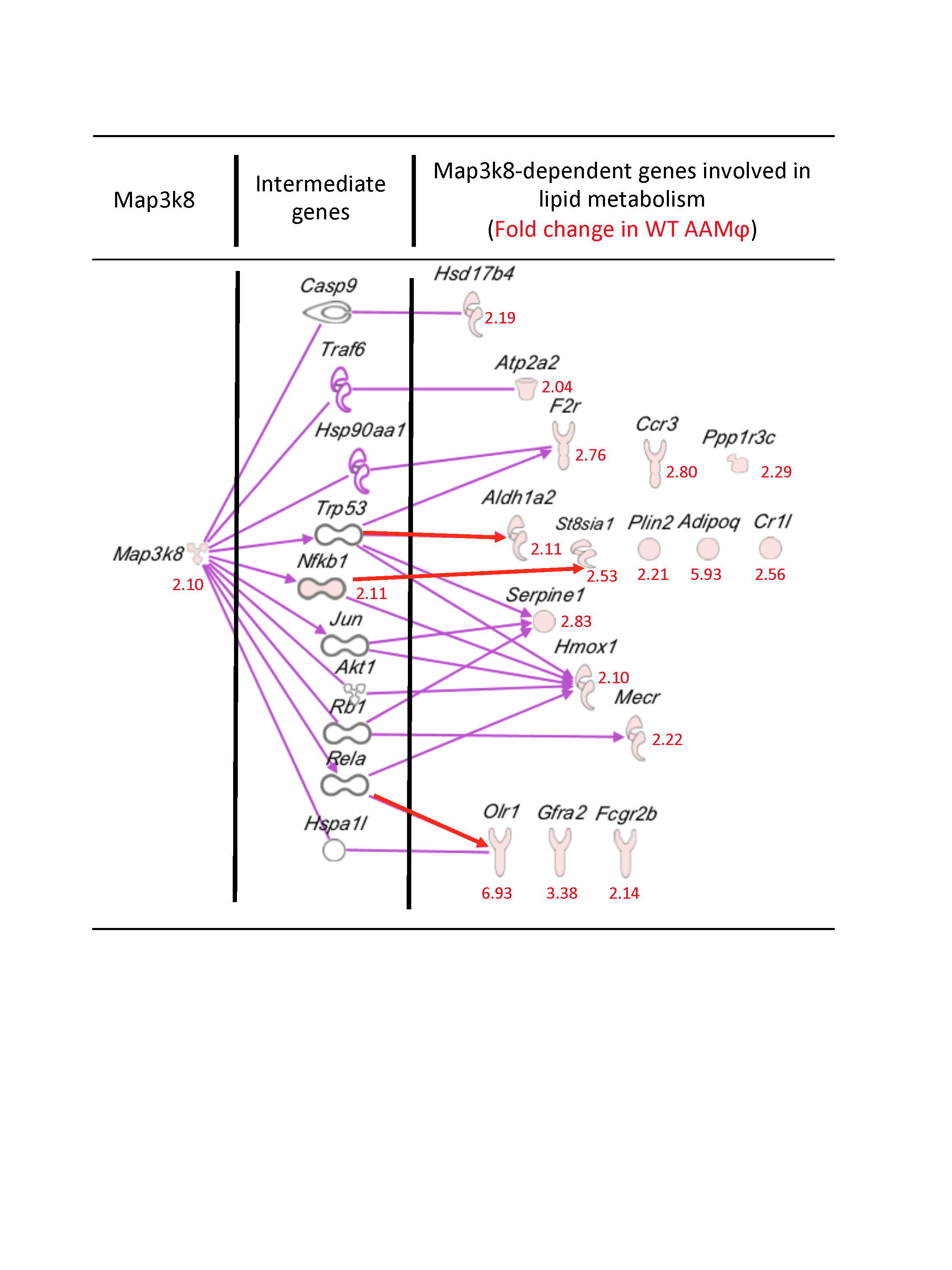

Supplement: S5 Fig — Ingenuity pathways analysis of lipid metabolism pathways (S1 Table) from bone marrow-derived macrophages (BMDM) stimulated with IL-4 and IL-13 for 24 hours, as in Figs 5 and 6. Elevated genes involved in lipid metabolism in WT, but not Map3k8 –/–macrophages, are displayed, with their relationship with Map3k8 highlighted via intermediate genes. (TIFF) [file ppat.1005783.s005.tiff]
