# Supplementary material for: TPL-2 Regulates Macrophage Lipid Metabolism and M2 Differentiation to Control TH2-Mediated Immunopathology
Source: PLoS Pathog. 2016 Aug 3;12(8):e1005783. doi: 10.1371/journal.ppat.1005783 (PMC4972396; doi:10.1371/journal.ppat.1005783)
Supplement: S1 Table — Bone marrow-derived macrophages (BMDM) were stimulated with IL-4 and IL-13 for 24 hours, as in Fig 4. Analysis of genes involved in lipid metabolism was performed by Ingenuity pathways analysis. Gene expression is indicated, relative to un-stimulated macrophages. Highlighted genes are either absent in Map3k8 –/–M2 macrophages (Map3k8-dependent) or absent in both WT and Map3k8 –/–M2 macrophages. (PDF) [file ppat.1005783.s007.pdf]

	Fold Change (IL-4+IL-13/PBS)	
**Symbol**	**WT**	***Map3k8–/–***
**FLT1**	35.893	45.835
**PTGS1**	29.603	25.473
**GATM**	7.133	5.784
**OLR1**	6.918	0
**PPARGC1B**	6.388	6.462
**ACPP**	6.188	8.948
**ADIPOQ**	5.934	0
**APOE**	5.576	5.297
**PRKCD**	5.426	5.376
**FABP7**	5.385	5.163
**CD74**	5.329	6.127
**APOC2**	5.233	5.746
**CD36**	4.772	5.583
**CLEC7A**	4.701	5.063
**SLC36A2**	4.636	6.057
**IGF1**	4.627	5.553
**DAGLB**	4.574	5.974
**ADORA2B**	4.534	3.232
**CXCL14**	4.393	5.338
**GPC1**	4.3	8.26
**ANGPTL4**	4.209	3.733
**TCF7L2**	4.07	5.52
**TBXAS1**	4.023	4.504
**APEX1**	3.996	4.172
**FABP4**	3.788	3.027
**CEBPA**	3.711	4.828
**COTL1**	3.694	3.914
**UCP2**	3.577	4.051
**CYP1B1**	3.423	3.298
**NQO1**	3.38	2.724
**GFRA2**	3.377	0
**SH3KBP1**	3.336	3.573
**HTT**	3.188	2.765
**PGAP1**	3.061	3.52
**MAN2B1**	3.023	3.018
**C1QA**	2.91	2.714
**LPCAT2**	2.878	3.506
**PIKFYVE**	2.854	2.508
**SERPINE1**	2.835	0
**CCR3**	2.793	0
**ABCD2**	2.782	4.097
**ME1**	2.76	2.502
**GNAS**	2.759	3.346

**F2R**	2.754	0
**TGM2**	2.732	3.599
**ANG**	2.726	2.909
**OCRL**	2.678	2.633
**PPARG**	2.587	2.407
**ST8SIA1**	2.533	0
**IDH1**	2.521	2.836
**TNFRSF1B**	2.517	3.035
**CR1L**	2.507	0
**MITF**	2.478	2.654
**HGF**	2.465	2.693
**VAV2**	2.353	2.621
**ATF3**	2.346	2.098
**HPGD**	2.315	2.789
**Apoc1**	2.292	2.989
**CYP4F2**	2.292	2.863
**LRP1**	2.275	2.495
**LIMK1**	2.258	2.735
**ITGB3**	2.256	2.31
**MECR**	2.223	0
**PPP1R3C**	2.204	0
**SLC13A3**	2.19	2.227
**HSD17B4**	2.188	0
**PDGFB**	2.179	2.773
**RILP**	2.177	2.365
**CPT1A**	2.176	2.67
**PDGFA**	2.145	2.516
**PLA2G15**	2.145	2.59
**FCGR2B**	2.144	0
**EDN1**	2.112	2.252
**ALDH1A2**	2.107	0
**C3**	2.1	2.644
**HMOX1**	2.098	0
**PLA2G5**	2.094	3.18
**TIMP1**	2.086	2.062
**PARP1**	2.081	2.293
**NFKB1**	2.044	2.012
**ATP2A2**	2.036	0
**JAK2**	2.032	2.134
**RXRA**	2.028	2.743
**PDIA3**	2.023	2.088
**RUNX1**	2.022	2.107
**CXCL12**	2.02	2.055
**MAP3K8**	2.013	2.013
**PTGER2**	2.013	2.005
**PLIN2**	2.003	0

PDE3B	2.001	2.272
XPA	-2.019	0
KIT	-2.029	-2.193
SMPD1	-2.035	0
SMAD3	-2.04	0
FAM213B	-2.05	0
STARD4	-2.058	0
CD81	-2.061	0
FDFT1	-2.062	-2.157
ABCB1	-2.064	0
SREBF2	-2.066	0
ABCB4	-2.093	0
CCL5	-2.093	0
NEU1	-2.097	0
CRY1	-2.1	-2.038
GPD2	-2.122	0
HCAR2	-2.129	-2.517
CAV2	-2.151	-2.082
SRGAP3	-2.163	-2.444
CEBPB	-2.163	0
MGST2	-2.189	-2.286
CDKN1B	-2.225	-2.227
IL1B	-2.225	0
FADS1	-2.261	0
CDKN2C	-2.262	-2.351
ADIPOR1	-2.269	0
NFKBIA	-2.271	-2.361
PANK1	-2.291	0
KDR	-2.297	-2.153
BCL2	-2.372	-2.286
CDKN1A	-2.38	-2.31
FCGR1A	-2.394	-2.464
MIF	-2.408	-2.518
IL1RN	-2.41	-2.087
ACOT13	-2.412	-2.24
C5AR1	-2.422	-2.021
LSS	-2.439	-2.181
PTTG1	-2.439	-2.048
TLR2	-2.449	-2.045
RRAD	-2.476	-2.315
ELOVL7	-2.511	-3.899
CHPT1	-2.514	-2.157
PPAP2C	-2.55	-2.369
PDPN	-2.562	-2.425
CFH	-2.573	-2.098
ENTPD5	-2.583	-2.173

CD82	-2.601	-2.259
IGFBP4	-2.618	0
HMGA1	-2.618	0
DHCR24	-2.628	-2.619
RGS2	-2.63	-2.612
PLSCR1	-2.632	-2.352
SC5D	-2.647	-2.616
B4GALT6	-2.771	-2.232
UTP14C	-2.784	-2.352
ASAH2	-2.825	-2.64
Acot1	-2.874	-2.28
ACER3	-2.894	-3.022
ACOT2	-2.919	-2.857
ACSS2	-2.947	-2.791
IL16	-2.948	-3.217
CERS4	-2.954	-3.141
BCL2L1	-2.956	-2.512
INSIG1	-2.995	-2.825
EDNRB	-3.016	-4.243
DBP	-3.045	-2.7
BID	-3.054	-3.028
Abcb1b	-3.057	-2.463
LDLR	-3.067	-3.086
IDI1	-3.085	-2.779
IL10	-3.193	-3.71
ANGPTL6	-3.221	-3.486
ACSL4	-3.298	-2.687
LTC4S	-3.328	-3.121
SGMS2	-3.342	-2.625
FPR2	-3.378	-5.362
HPGDS	-3.394	-3.968
CSF1	-3.414	-2.186
FADS2	-3.447	-3.981
IL1R2	-3.472	-4.238
IL18	-3.485	-2.97
ALOX5	-3.593	-2.729
ITGA6	-3.609	-3.174
CYSLTR1	-3.66	-3.705
RORA	-3.761	-3.047
PDCD6IP	-3.813	-3.638
ETNK1	-3.882	-3.928
CEACAM1	-3.967	-3.644
IL6	-3.975	0
PDE8B	-4.058	-3.331
ETV1	-4.063	-4.216
SLC38A2	-4.099	-3.71

CDKN2D	-4.145	-4.472
PIK3CG	-4.181	-3.253
SNCAIP	-4.285	-4.752
UGCG	-4.285	-3.985
AGPAT9	-4.586	0
CCL2	-4.792	-6.091
IGHM	-4.844	-3.892
SOAT2	-4.862	-7.045
PRKG1	-5.234	-2.182
FOS	-5.353	-5.65
CXCL10	-5.354	-4.918
MID1IP1	-5.36	-5.025
ACSL3	-5.481	-3.764
P2RY13	-5.595	-5.142
ESR1	-5.814	-4.621
BNIP3	-5.91	-5.93
OSM	-5.944	-5.481
ST8SIA4	-5.951	-5.033
DIO2	-6.375	-4.898
PLAU	-6.411	-6.634
ADRB2	-6.942	-4.87
Scd2	-7.267	-5.613
PCK2	-7.38	-7.006
Ccl2	-7.488	-6.578
NR4A1	-7.539	-5.195
NR4A2	-7.628	-6.322
SCD	-7.781	-8.574
EGR1	-7.852	-5.619
CPE	-8.66	-5.74
CD14	-9.57	-13.044
DUSP1	-9.641	-9.284
AQP9	-14.304	-18.366
VEGFA	-15.278	-12.467
SOCS3	-20.035	-20.808
PHGDH	-20.826	-22.684
GDF15	-23.322	-31.973
TRIB3	-29.442	-27.771
PTGS2	-30.98	-29.264
